# Preclinical-to-clinical Anti-cancer Drug Response Prediction and Biomarker Identification Using TINDL

**DOI:** 10.1016/j.gpb.2023.01.006

**Published:** 2023-02-11

**Authors:** David Earl Hostallero, Lixuan Wei, Liewei Wang, Junmei Cairns, Amin Emad

**Affiliations:** 1 Department of Electrical and Computer Engineering, McGill University, Montreal, QC H3A, Canada; 2 Mila – Quebec Artificial Intelligence Institute, Montreal, QC H2S, Canada; 3 Department of Molecular Pharmacology and Experimental Therapeutics, Mayo Clinic, Rochester, MN 55905, USA; 4 The Rosalind and Morris Goodman Cancer Institute, McGill University, Montreal, QC H3A, Canada

**Keywords:** Drug response, Deep learning, Explainable AI, Cancer, Gene knockdown experiment

## Abstract

Prediction of the response of **cancer** patients to different treatments and identification of biomarkers of **drug response** are two major goals of individualized medicine. Here, we developed a **deep learning** framework called TINDL, completely trained on preclinical cancer cell lines (CCLs), to predict the response of cancer patients to different treatments. TINDL utilizes a tissue-informed normalization to account for the tissue type and cancer type of the tumors and to reduce the statistical discrepancies between CCLs and patient tumors. Moreover, by making the deep learning black box interpretable, this model identifies a small set of genes whose expression levels are predictive of drug response in the trained model, enabling identification of biomarkers of drug response. Using data from two large databases of CCLs and cancer tumors, we showed that this model can distinguish between sensitive and resistant tumors for 10 (out of 14) drugs, outperforming various other machine learning models. In addition, our small interfering RNA (siRNA) knockdown experiments on 10 genes identified by this model for one of the drugs (tamoxifen) confirmed that tamoxifen sensitivity is substantially influenced by all of these genes in MCF7 cells, and seven of these genes in T47D cells. Furthermore, genes implicated for multiple drugs pointed to shared mechanism of action among drugs and suggested several important signaling pathways. In summary, this study provides a powerful deep learning framework for prediction of drug response and identification of biomarkers of drug response in cancer. The code can be accessed at https://github.com/ddhostallero/tindl.

## Introduction

Cancer is one of the deadliest public health problems worldwide, and cases are still rapidly growing. In 2020, it is estimated that around 10 million people have died of cancer [1]. Individualized medicine is a promising concept, which aims to improve the prognosis of patients by adapting the patient’s treatment to their unique clinical and molecular characteristics. One of the main goals of individualized medicine is the prediction of the response of patients to different treatments, and identification of biomarkers that enable such prediction. High-throughput sequencing technologies along with major initiatives such as The Cancer Genome Atlas (TCGA) [2] have provided a unique opportunity for machine learning (ML) algorithms to address these challenges. However, ML models and particularly deep learning (DL) approaches require a large number of samples with known drug response to train generalizable models. However, data on clinical drug response (CDR) of cancer patients, even in large databases such as TCGA, are usually small for most drugs and do not lend themselves to the training of DL models.

On the other hand, large databases of molecular profiles of hundreds of *in vitro* cancer cell lines (CCLs) and their response to hundreds of drugs [3], [4], [5] have enabled development of various ML algorithms for prediction of drug response [6], [7], [8]. Unfortunately, these models, even though accurate in predicting the drug response of held-out CCLs, usually do not generalize well to predicting the CDR of real tumors from cancer patients, and their prediction performance significantly deteriorates due to the major biological and statistical differences between CCLs and tumors [9].

Recognizing these issues, some studies have adopted to utilize tumor samples with known CDR in the training of their models, either by fully training their models on data corresponding to tumor samples [10], [11], [12], or using them in addition to CCLs (*e.g.*, using transfer learning [13]). However, as a result of this strategy, these studies have only been able to develop models on very few drugs due to the small sample sizes of patient cohort data with known drug response. Another strategy is to train ML models completely on preclinical CCLs but use computational approaches to overcome the statistical differences between CCLs and tumors. For example, multiple approaches [9], [14] have used batch removal methods such as ComBat [15] to reduce the discrepancy between the training CCLs and test tumors. One limitation of these methods is that ComBat is used as a preprocessing step such that the gene expression (GEx) profiles of both CCLs (training set) and tumors (test set) are adjusted. As a result, prediction of CDR of new cancer patients requires retraining of the model.

In this study, our goal was to develop a DL computational pipeline, fully trained on the GEx profile and drug response of preclinical CCLs, to (1) predict the CDR of cancer patients and (2) identify biomarkers of drug response for a variety of cancer drugs. Motivated by Huang et al. [9], who showed that carefully incorporating information on the tissue (or cancer) types of the test samples can improve the predictive power of computational models, we developed a DL pipeline with tissue-informed normalization (TINDL) to achieve these goals. Unlike methods mentioned above, TINDL requires normalization of only test samples, and as a result retraining of the model is not necessary for new test samples.

The TINDL pipeline includes two phases. The first phase is responsible for prediction of CDR of cancer patients, and the second phase makes these predictions interpretable by identifying a small number of genes that considerably contribute to the predictive ability of the model. Focusing on drugs shared between the Genomics of Drug Sensitivity in Cancer (GDSC) [3] and TCGA [2], we showed that TINDL can distinguish between the sensitive and resistant patients for 10 (out of 14) drugs, considerably improving the performance of other methods, including our previous work, tissue-guided least absolute shrinkage and selection operator (TG-LASSO) [9]. TINDL utilizes a simple, yet effective, tissue-informed normalization to reduce the statistical discrepancies between the GEx profiles of the training and test samples. We showed that TINDL outperforms other DL-based models that try to explicitly remove these discrepancies using other techniques such as ComBat or domain adaptation [16], [17].

Focusing on tamoxifen, for which TINDL performed best, we showed that only a small panel of genes identified by TINDL can be used to predict the CDR of cancer patients. Moreover, using small interfering RNA (siRNA) gene knockdown of 10 genes identified by TINDL in two breast CCLs (MCF7 and T47D), we showed that the knockdown of any of these genes significantly changed the response to tamoxifen in MCF7 and the knockdown of 7 of them significantly changed the response to this drug in T47D. These *in vitro* experiments further validated the TINDL pipeline and its ability to identify biomarkers of drug response.

## Results

### Prediction of CDR and identification of biomarkers of drug response using cell line data

**Figure 1:**
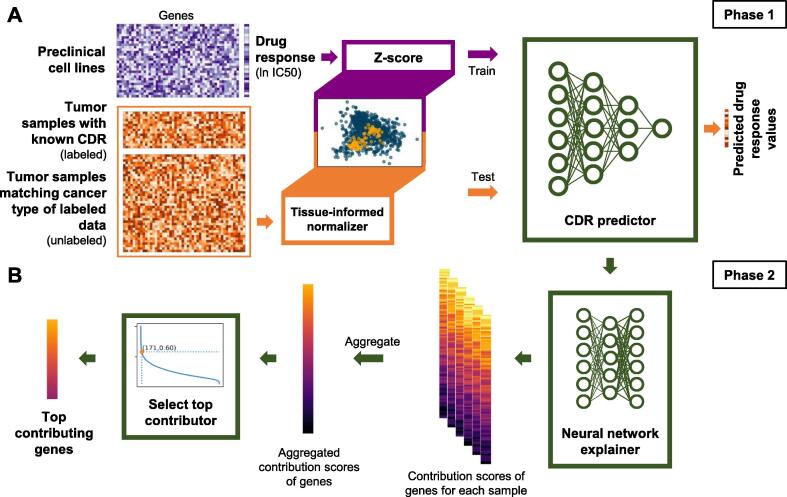
**The pipeline used for prediction of drug responses and identification of important genes** **A.** In phase 1, the gene expression data of the CCLs and ln IC50 values were both z-score normalized, whereas the tumor gene expression data (test data) were normalized using the tissue-informed normalizer. We then used this model to train a CDR predictor using the CCL data. After training, the model predicted the drug response value for the tumors. **B.** In phase 2, the trained CDR predictor was used to train a neural network explainer using the same training data. We used the trained explainer to give gene contribution scores for each genes of the test samples. We aggregated the scores across samples and then selected the top genes by estimating the point of maximum curvature. CCL, cancer cell line; CDR, cancer drug response; IC50, half-maximal inhibitory concentration.

We developed TINDL to (1) predict the CDR of cancer patients (test set) and (2) identify predictive biomarkers of drug response based on models completely trained on preclinical cell line data (training set). The pipeline has two major phases: the modeling phase and the gene identification phase. In the modeling phase (Figure 1A), a neural network is trained using the GEx profiles of CCLs and their response to a drug [*i.e.*, normalized ln IC50 values in this study, where IC50 stands for half-maximal inhibitory concentration]. The trained model was then used to predict the drug response of cancer patients based on the carefully normalized GEx profiles of their primary tumors. Details of the DL architecture are provided in Materials and methods.

We designed the normalization step of GEx profiles of patient tumors to address two important issues. First, we required this approach to remove the discrepancy between the statistical properties of GEx of CCLs and patient tumors, originating from the technical differences in protocols for measuring the data and the biological differences between preclinical CCLs and clinical tumors. Second, we required this approach to incorporate information on the tissues of origin (or cancer types) of tumors in the prediction task. In a previous study [9], we showed that information on the tissues of origin of samples plays an important role in improving prediction performance; however, most commonly used methods for this task are not capable of appropriately incorporating this information. For this purpose and given a drug, we first identified the set of tissues (henceforth referred to as “target tissues”) of the clinical samples to which the drug was administered. Then, we collected additional GEx profile of samples from the same target tissues, independent of what drug was used for their treatment. The GEx profile of each test sample was then normalized against this additional set of “unlabeled” data (see Materials and methods for details).

This simple, yet effective, normalization approach used in our pipeline removes the statistical discrepancy between the test and training datasets by mapping the expression of each gene in each dataset to a distribution with unit variance and zero mean. However, because the test samples are normalized while considering the GEx of a much larger unlabeled set of samples, this normalization will not be negatively affected if the size of the test set is small (*e.g.*, if we want to predict the drug response of a single sample), which is superior compared with methods that perform the normalization using only the test samples. In addition, because the normalization is done independently for the training and test sets, one does not need to retrain the DL model every time in which the drug response of a new test sample is to be predicted (a shortcoming of our previous approach [9]).

The second phase of the pipeline seeks to assign a contribution score to each gene based on its contribution to the trained predictive model to enable interpretability of the model (Figure 1B). In this phase, we first used CXPlain [18] to assign a sample-specific score to each gene. These scores were then averaged over all samples (separately for each gene) and normalized to provide a final contribution score. Additionally, we used the distribution of these scores to systematically identify the critical point that the contribution of the genes diminishes, enabling us to narrow down the top ranked list of genes for follow-up analysis (pathway enrichment analysis, gene knockdown experiments, *etc*.). The details of this phase are provided in Materials and methods.

### TINDL distinguishes between sensitive and resistant patients for the majority of the evaluated drugs

In order to assess the performance of TINDL in predicting CDR of cancer patients, we obtained GEx profiles of primary cancer tumors from the TCGA database [2]. We used the data corresponding to Response Evaluation Criteria in Solid Tumors (RECIST) CDR of TCGA patients, collected and processed in a previous study [10], and identified 14 drugs that satisfied two conditions: (1) there were at least 20 patients with known CDR values for each drug in TCGA database and (2) the ln IC50 drug response values of these drugs were measured in the GDSC database. Similar to previous studies [9], [14], we transformed the CDR of these tumors into a Boolean label in which “resistant” referred to patients with CDR of “stable disease” or “progressive disease” and “sensitive” referred to patients with CDR of “complete response” or “partial response”. These CDR values were used to evaluate the predicted drug response values using TINDL and other algorithms but were not used for training them. The list of these 14 drugs, number of TCGA patients, and their cancer types are provided in Table S1. Similarly, we obtained GEx profiles and ln IC50 drug response values of CCLs from different lineages from the GDSC database [3], corresponding to the 14 drugs mentioned above (see Table S1 for the number of training samples for each drug).

Following previous work in this area [9], [14], we used a one-sided Mann–Whitney *U* test to determine if the predicted ln IC50 values of resistant patients for a drug are significantly higher than those of sensitive patients. Table 1, Table S2, and Figures S1 and S2 show the performance of TINDL in the prediction of CDR of TCGA samples using preclinical GDSC samples for different drugs. TINDL is capable of distinguishing between resistant and sensitive patients for 10 (out of 14) drugs (*P* < 0.05, one-sided Mann–Whitney *U* test) with a combined *P* value of 2.77E–10 (Fisher’s method).

**Table 1:** **The number of TCGA samples and the performance of TINDL in predicting their CDR for 14 drugs**

**Drug**	**Number of clinical samples**	**Number of sensitive samples**	**Number of resistant samples**	***P* value**
Cisplatin	303	237	66	6.36E−4
Tamoxifen	20	14	6	1.14E−3
Etoposide	84	73	11	4.00E−3
Doxorubicin	100	68	32	1.42E−2
Paclitaxel	158	111	47	2.29E−2
Vinorelbine	30	23	7	2.41E−2
Oxaliplatin	54	33	21	2.41E−2
Temozolomide	95	11	84	2.94E−2
Bleomycin	52	46	6	3.41E−2
Gemcitabine	157	75	82	4.57E−2
Cyclophosphamide	101	96	5	5.60E−2
Pemetrexed	38	18	20	2.86E−1
Irinotecan	23	6	17	3.04E−1
Docetaxel	102	67	35	7.04E−1

*Note*: *P* values were calculated by a one-sided Mann–Whitney *U* test to determine if TINDL can distinguish between sensitive and resistant patients. To ensure the results are not biased by the initialization of the parameters of model, TINDL was trained using ten random initializations, and the mean aggregate of its prediction was used to calculate the *P* values. Drugs were sorted based on their associated *P* values. TINDL, deep learning pipeline with tissue-informed normalization; TCGA, The Cancer Genome Atlas; CDR, cancer drug response.

**Figure 2:**
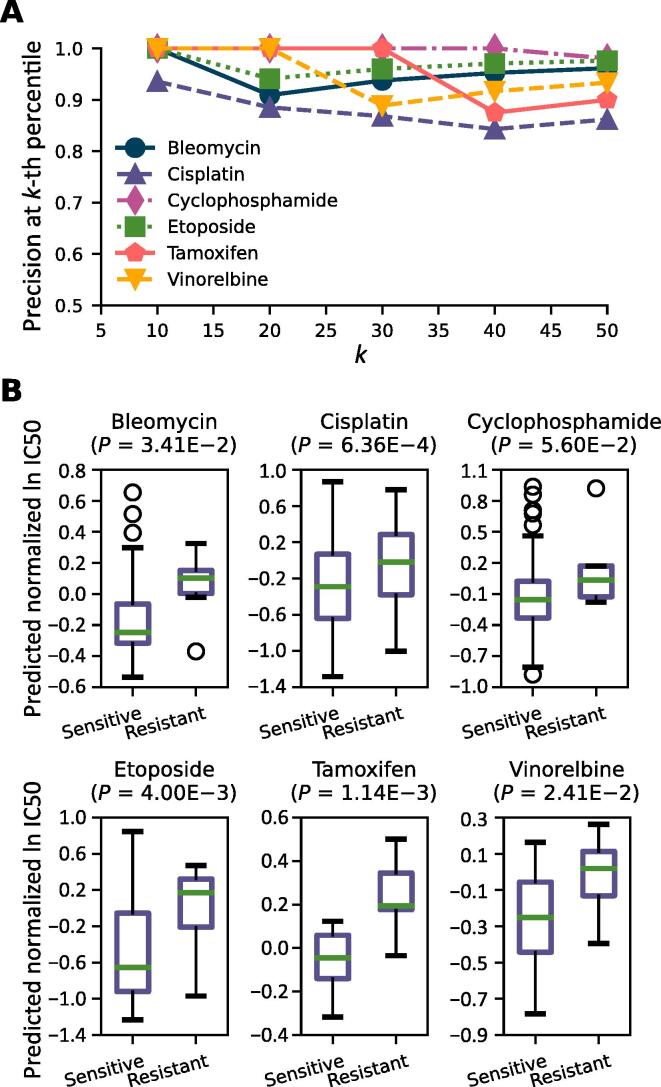
**Performance metrics for a subset of the drugs** To prevent the figure from becoming cluttered, the results corresponding to only six drugs are shown (see Tables S2 and S3 for performance metrics of all drugs). **A.** Precision at *k*-th percentile for identification of sensitive patients. **B.** Distribution of predicted drug response for sensitive and resistant patients. The *P* values are calculated using a one-sided Mann**–**Whitney *U* test.

Next, we defined a measure called precision at *k*-th percentile to determine whether patients whose predicted ln IC50 is within the lower tail of the distribution correspond to sensitive patients (*i.e.*, responders to the drug). For different values of *k*, tumors with predicted ln IC50 in the bottom *k*% were predicted as sensitive, and their count was used to calculate precision. Figure 2A and Table S3 show precision at *k*-th percentile of TINDL for different values of *k*. These results suggest that for six drugs (tamoxifen, etoposide, vinorelbine, cyclophosphamide, bleomycin, and cisplatin), TINDL can identify responders with a precision at *k*-th percentile above 84% for any choice of *k*. The distribution of predicted CDR values for sensitive and resistant patients for these drugs are shown in Figure 2B.

### TINDL outperforms alternative methods in prediction of CDR

Next, we sought to determine how TINDL performs against alternative computational models. For this purpose, we considered multiple traditional and state-of-the-art ML models [9], [14] for predicting CDR of cancer patients from preclinical CCLs. The detailed performance measures for each drug and each model are provided in Table S2 and Figures S1 and S2, and the summary of the results are provided in Table 2. In this table, we used the combined *P* value of 14 drugs to summarize the performance of different methods (Fisher’s method). As shown in Table 2, TINDL can distinguish between sensitive and resistant patients for 10 (out of 14) drugs (with a combined *P* value of 2.77E−10 for all drugs), whereas the second-best method in this table can only distinguish between sensitive and resistant patients for 7 drugs. Similar to our previous study [9], we also observed that regression with least absolute shrinkage and selection operator (LASSO) and its variation, TG-LASSO, performed reasonably well (when considering all drugs), whereas support vector regression (SVR) and random forests did not perform as well.

**Table 2:** **The performance of different computational models in predicting CDR of TCGA samples using models completely trained on preclinical GDSC CCLs**

**Algorithm**	**Number of drugs with *P* < 0.05(one-sided Mann–Whitney *U* test)**	**Total number of evaluated drugs**	**Combined *P* value (Fisher)**
TINDL	10	14	2.77E−10
LASSO	7	14	7.47E−7
TG-LASSO [9]	6	14	8.32E−7
SVR (RBF kernel)	5	14	1.89E−6
Geeleher, et al. [14]	4	14	5.63E−3
Random forests	4	14	3.12E−3

*Note*: The combined *P* value combined over all 14 drugs using Fisher’s method. CCL, cancer cell line; GDSC, Genomics of Drug Sensitivity in Cancer; LASSO, least absolute shrinkage and selection operator; SVR, support vector regression; TG-LASSO, tissue-guided LASSO.

As discussed earlier, one of the major challenges in predicting the CDR of cancer patients based on ML models trained on preclinical CCLs is the statistical differences between these samples. To assess the performance of TINDL against other DL models that explicitly try to remove these statistical differences, we considered three alternative methods, as well as two baselines that could be considered “default workflows”, had we not foreseen the dire impact of these statistical differences. The first method (referred to as ComBat-DL) utilizes ComBat [15] as a preprocessing step to remove the statistical discrepancy between CCLs and tumor samples. ComBat [15] is a popular method for removing batch effects in GEx datasets and has been widely used for drug response prediction [9], [14], [19] and other applications [20], [21]. The ComBat-transformed GEx profiles are then used in a DL architecture similar to TINDL for a fair comparison. The second and third methods are based on Domain Adaptive Neural Network (DANN) [16] and Adversarial Discriminative Domain Adaptation (ADDA) [17], two domain adaptation techniques that were originally developed for image processing, so here we called them DANN-DL and ADDA-DL, respectively. Instead of adapting the GEx input features, these methods adjust the latent feature representations learned by the encoder. DANN uses adversarial neural networks to create a shared latent feature space between the datasets. ADDA, on the other hand, is a unidirectional domain adaptation approach that builds over a pretrained predictor and tries to adapt the first few layers of the neural network such that the latent feature representation of target dataset aligns with that of the source dataset.

Although the three approaches mentioned above actively try to reduce the discrepancy between the training set and test set, two default workflows (TrainNorm-DL and TestNorm-DL) actively ignore this challenge. In particular, TrainNorm-DL assumes that the test set (tumors) comes from the same distribution as the training set (CCLs), and therefore uses the mean and standard deviation of the training set to normalize all of the data. This is essentially the default workflow for most ML tasks in order to prevent data leakage during normalization. The TestNorm-DL normalizes the test set and training set separately (*i.e.*, it uses the mean and standard deviation of the test set to normalize itself). One should note that TestNorm-DL is not an ideal approach in practice, because it requires a large number of test samples to be present and is not recommended when predicting the response of a small number of samples.

We trained models of these methods with a similar architecture to that of TINDL, with the exception of the discriminators, which are specific to ADDA and DANN and are used for domain adaptation. The details of these methods, including their architecture and training procedure, are provided in Materials and methods and File S1. Table 3 and Table S2 show the performance of these DL-based approaches. These results showed that in all three cases of explicit discrepancy removal, only for 7 (out of 14) drugs the predicted normalized ln IC50 of sensitive patients was significantly smaller than those of resistant patients. As expected, TrainNorm-DL did not perform as well (6 out of 14) as the others DL approaches. TestNorm-DL was able to segregate sensitive patients in 8 drugs, which surprisingly came second to TINDL, but this method is not well suited for applications in which only very few samples exist in the test set.

**Table 3:** **The performance of DL-based methods that explicitly try to remove discrepancy between preclinical training and clinical test datasets**

**Algorithm**	**Number of drugs with*P*** **< 0.05 (a one-sided Mann–Whitney** ***U*** **test)**	**Tatol number of evaluated drugs**	**Combined*****P*****value (Fisher)**
ComBat-DL	7	14	6.73E−10
ADDA-DL	7	14	2.16E−7
DANN-DL	7	14	1.66E−6
TrainNorm-DL	6	14	4.68E−7
TestNorm-DL	8	14	1.80E−9

*Note*: The combined *P* value combined over all 14 drugs using Fisher’s method. To ensure a fair comparison, a similar architecture to TINDL was used for all these methods. Additionally, each model was trained using ten random initializations, and the mean aggregate of these predictions was used for calculating the *P* values. DL, deep learning; DANN, Domain Adaptive Neural Network; ADDA, Adversarial Discriminative Domain Adaptation.

**Figure 3:**
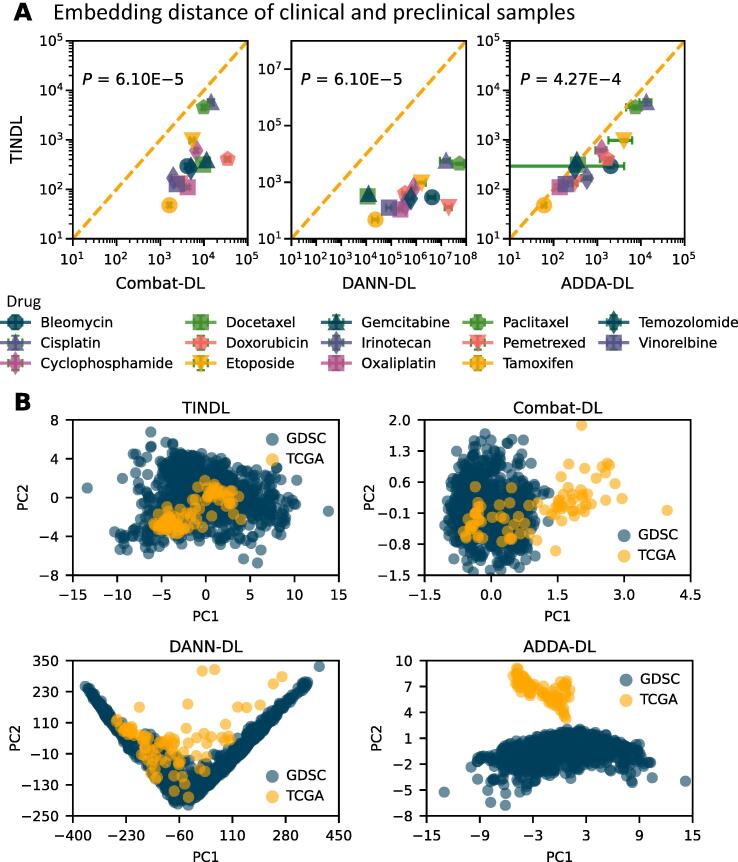
**Evaluation of the embeddings ofDLmodels** **A.** Scatter plots comparing the distance between preclinical and clinical samples in the embedding space for each drug. Each point in the scatter plot corresponds to a different drug. The *P* values are calculated using a one-sided Wilcoxon signed-rank test. The error bars show the 95% confidence intervals and are calculated based on ten runs of each method with random initializations. **B.** PCA of the embeddings used by each method to predict the response to etoposide. Visually, the TCGA samples are better mixed (*i.e.*, are not easily separable) with GDSC samples in TINDL compared with other methods. TINDL, deep learning pipeline with tissue-informed normalization; PCA, principal component analysis; PC, principal component; TCGA, The Cancer Genome Atlas; GDSC, Genomics of Drug Sensitivity in Cancer; DL, deep learning; DANN, Domain Adaptive Neural Network; ADDA, Adversarial Discriminative Domain Adaptation.

To assess the superior performance of TINDL compared with the first three DL-based models above, we assessed their ability in removing the discrepancy between preclinical and clinical samples. We did not include the default workflows in this analysis, because they ignore this discrepancy. For this purpose, we assessed the distance of clinical samples and preclinical samples for each method and each drug (see Materials and methods for details of calculating distances). Because methods that use domain adaptation do not modify the input features, but rather seek to remove the domain discrepancies in the latent space (the output of the encoder), we used the learned representation of each sample in the latent space for all methods. Using a one-sided Wilcoxon signed-rank test, we observed that the learned representations of TINDL for clinical samples have a significantly smaller average distance to preclinical samples compared with ComBat-DL (*P* = 6.10E−5), ADDA-DL (*P* = 4.27E−4), and DANN-DL (*P* = 6.10E−5), for all drugs (Figure 3A). The effectiveness of tissue-informed normalization of TINDL in removing the statistical discrepancy between the preclinical and clinical embeddings can also be visually observed using principal component analysis (PCA) and Uniform Manifold Approximation and Projection (UMAP) plots of the representations learned by each method (Figure 3B, Figures S3–S6).

Next, we sought to determine whether the latent space representation similarity has an influence on drug response prediction performance of TINDL across different drugs. We observed a negative Spearman rank correlation (r =  −0.17, *P* = 3.93E−2) between the aforementioned distances and the area under the receiver operating characteristic curve (AUROC) of prediction for different drugs. In particular, tamoxifen that had the highest AUROC (Table S2, AUROC = 0.92) also had the smallest average distance between clinical and preclinical representations of its samples among all drugs in TINDL. These results further support the conclusion that reducing the discrepancy between the statistical characteristics of clinical and preclinical samples plays an important role in the success of TINDL in the prediction of CDR.

### More complex neural network architectures do not show improvement

We also assessed the performance of different neural network architectures when used as the feature extractor, instead of fully-connected (FC) networks that were used in the previous section. Specifically, we used long short-term memory (LSTM), graph convolutional network (GCN) [22], and graph attention network (GAT) [23] for the first few layers of the model (see Materials and methods for details). All models were subjected to the same protocol and evaluation techniques as the other DL methods based of FC networks. A summary of the results are provided in Table 4, and more detailed evaluation metrics are provided in Table S2. Although in theory GCN and GAT may hold some advantage compared with a FC architecture because the features (GEx) are not independent, these architectures did not show an improvement over FC networks. LSTM was expected not to perform well because the data are not sequential. Nevertheless, it is interesting that for some of the drugs, the LSTM is able to separate the sensitive and resistant patients.

**Table 4:** **The performance of different neural network architectures when used as feature extractors**

**Architecture**	**Number of drugs with*P*** **< 0.05 (a one-sided Mann–Whitney** ***U*** **test)**	**Total number of evalutated drugs**	**Combined *P* value (Fisher)**
GAT	7	14	2.75E−11
GCN	6	14	2.85E−7
LSTM	6	14	1.86E−5

*Note*: The combined *P* value combined over all 14 drugs using Fisher’s method. To ensure a fair comparison, a similar architecture to TINDL was used for all these methods. Additionally, each model was trained using ten random initializations, and the mean aggregate of these predictions was used for calculating the *P* values. GAT, graph attention network; GCN, graph convolutional network; LSTM, long short-term memory.

### TINDL identifies biomarkers of drug response

We used TINDL (Figure 1B) to assign a score to the contribution of each gene in the trained model (see Material and methods for details). Figure S7 shows the distribution of these scores for each drug. To identify the threshold below which the contribution of the genes to the predictive model is small, we used a method called kneedle [24], which systematically determines this threshold for each drug based on the distribution of the scores. This method identified between 64 (for pemetrexed) to 243 (for bleomycin) genes, depending on the drugs. The ranked list of genes identified by TINDL using this drug-specific threshold is provided in Table S4.

Next, we sought to determine whether the identified genes are drug specific. To this end, we calculated the Jaccard similarity coefficient of drug pairs (Figure S8A). The results revealed a high degree of drug specificity with the average Jaccard similarity coefficient for all drugs equal to only 0.027. However, some genes were implicated for multiple drugs (Figure S8B; Table S5). Previous studies have shown that these genes are involved in several cancers and are associated with sensitivity to multiple drugs [25], [26], [27], [28], [29], [30], [31]. Multidrug resistance (MDR) is one of the reasons for reduced effectiveness of many cancer therapeutic agents [32]. MDR is defined as the insensitivity to therapeutic substances that are not associated by structure or mechanism of action [33]. The classical mechanism of MDR is associated with the overexpression of the ATP-binding cassette (ABC) transporter genes (*ABCB1*, *ABCD1*, *etc*.), which contribute to the reduction of the effective drug concentration transporting the drug out of the cells [34]. In addition to the classical MDR mechanism associated with the overexpression of ABC genes, there are atypical mechanisms [35], [36], [37]. Examples of these atypical mechanisms include escaping adaptive immune responses [35]. Dysregulation of many genes, *e.g.*, *APOBEC3A*, promote evolution and progression of cancers, escape adaptive immune responses, and lead to development of drug resistance in multiple cancers [38], [39]. Other atypical mechanisms include dysregulation of genes, such as *CRYAB*, related to macrophage infiltration and polarization [36], and dysregulation of genes that regulate drug-induced apoptosis by activating the survival pathways such as MEK/ERK signaling and inhibiting the mitochondrial apoptosis pathway in cervical cancer cells [37]. In particular, Schlafen family member 11 (SLFN11) was implicated for nine drugs and was the top contributor for bleomycin, cisplatin, doxorubicin, etoposide, gemcitabine, and irinotecan, and the top third contributor for oxaliplatin. SLFN11 is a putative DNA/RNA helicase that is recruited to the stressed replication fork and inhibits DNA replication. DNA replication is one of the fundamental biological processes in which dysregulation can cause genome instability [40]. This instability is one of the hallmarks of cancer and confers genetic diversity during tumorigenesis [41]. Various studies have shown that the expression of this gene sensitizes cancer cells to many chemotherapeutic agents including cisplatin, oxaliplatin, irinotecan, gemcitabine, doxorubicin, and etoposide [42], [43], [44]. Epigenetically mediated suppression of SLFN11 via EZH2 contributes to acquired chemotherapy resistance, one that can be prevented and/or actively remodeled through targeting EZH2 [45]. Several potent and selective EZH2 inhibitors are now in different stages of clinical development with promising safety profile, including phase II (Epizyme) and phase I (Constellation, GSK) trials in multiple solid tumor and hematological indications. Our data support the notion that the combination of down-regulating SLFN11 via EZH2 inhibitor with chemotherapeutic reagents should be considered in multiple cancer types [46].

To better understand the functional characteristics of genes implicated by TINDL for multiple drugs, we used KnowEnG’s gene set characterization (GSC) pipeline [47] to identify pathways associated with 29 genes identified by TINDL for at least 4 drugs (Figure S8B). This pipeline enables identification of associated pathways while incorporating interactions among genes and their protein products through network-guided analysis. The results (Table S5) implicated five pathways, including “regulation of toll-like receptor signaling pathway”, “alpha-synuclein signaling”, “Arf6 trafficking events”, “insulin pathway”, and “RalA downstream regulated genes”. Innate immune receptors such as toll-like receptors (TLRs) are responsible for recognizing molecular patterns associated with pathogens and provide critical molecular links between innate cells and adaptive immune responses. Engagement of TLRs on dendritic cells (DCs) promotes cross-talk between the innate and the adoptive immune system, maturation and migration of DCs into lymph nodes leading to activation, and proliferation and survival of tumor antigen-specific naïve CD4^+^ and CD8^+^ T cells [48]. Tumor cells themselves do not express molecules which would induce DC maturation, so application of TLR agonists is an important element of immunotherapy protocols aiming T cell activation [49]. In addition, TLR agonists have been proposed as adjuvants for cancer vaccines [50]. TLR3 agonist as an adjuvant with conventional chemotherapy can break tolerogenic or immunosuppressive effects generated by the tumor and drive T cell responses and tumor rejection [51], [52].

Alpha-synuclein (α-syn) is a neuronal protein responsible for regulating synaptic vesicle trafficking. α-syn is frequently expressed in various brain tumors and melanoma [53], and its up-regulation has been linked to aggressive phenotypes of meningiomas [54]. Moreover, loss of α-syn results in dysregulation of iron metabolism and suppression of melanoma tumor growth [55]. Oncogenic activation of synuclein contributes to the cancer development by promoting tumor cell survival via activation of JNK/caspase apoptosis pathway and ERK, and by providing resistance to certain chemotherapeutic drugs [56], suggesting synuclein as a new therapeutic target for future treatment to overcome resistance to certain chemotherapeutic. ADP-ribosylation factor 6 (ARF6) governs the trafficking of bioactive cargos to tumor-derived microvesicles (TMVs) which comprise a class of extracellular vesicles released from tumor cells that facilitate communication between the tumor and the surrounding microenvironment [57]. Invasive tumor cells shed TMVs containing bioactive cargo and utilize TMVs to degrade extracellular matrix during cell invasion [58]. Indeed, several studies have suggested a correlation between ARF6 expression and invasion and metastasis of multiple cancers [59], [60], suggesting that antagonistic ARF6 signaling can dictate TMV shedding and the overall mode of invasion. Insulin, a signaling molecule that controls systemic metabolic homeostasis, can be seen as enabling tumor development by providing a mechanism for PI3K activation and enhanced glucose uptake [61], [62], and plays a role in cytotoxic therapy response [63]. RAS-related protein RalA is a member of the Ral family, and the RalA pathway contributes to anchorage independent growth, tumorigenicity, migration, and metastasis [64], [65]. In conclusion, the link between genes implicated for multiple drugs and the pathways mentioned above that play different roles in cancer may point to shared mechanisms of action among different anti-cancer drugs. We also performed a similar pathway enrichment analysis for genes implicated for each drug separately and the results are provided in Table S6.

### Functional validation confirms the role of TINDL-identified genes in response to tamoxifen

**Figure 4:**
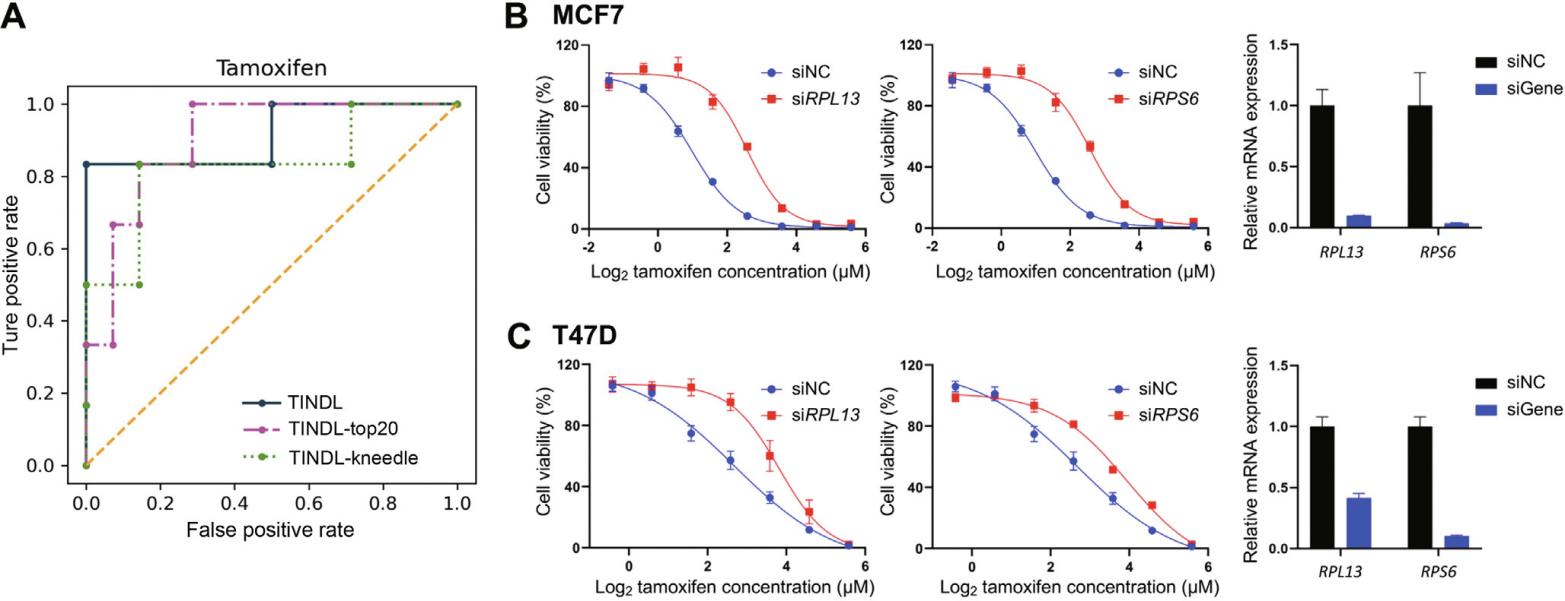
**Top genes identified for tamoxifenresponseand their functional validation** **A.** The ROC curves for tamoxifen when different number of genes were used for CDR prediction. TINDL utilized the GEx values of all genes (AUROC = 0.92), whereas TINDL-top20 (AUROC = 0.90) and TINDL-kneedle (AUROC = 0.83) assigned a value of 0 to all genes except for top 20 and top genes identified by kneedle, respectively. **B.** Tamoxifen dose–response curves corresponding to the siRNA knockdown of *RPS6* and *RPL13* in MCF7 cells. Cytotoxicity assays were performed using technical triplicate experiments with three wells per drug concentration. Knockdown efficiency was assessed by qRT-PCR using three technical replicates (Table S8). The dose–response curves for all genes are provided in Figure S9. **C.** Tamoxifen dose–response curves corresponding to the siRNA knockdown of *RPS6* and *RPL13* in T47D cells. Cytotoxicity assays were performed using technical triplicate experiments with three wells per drug concentration. Knockdown efficiency was assessed by qRT-PCR using three technical replicates (Table S8). The dose–response curves for all genes are provided in Figure S10. All *P* values are calculated using an extra sum-of-squares F test. ROC, receiver operating characteristic; AUROC, area under the receiver operating characteristic curve; GEx, gene expression; qRT-PCR, quantitative real-time polymerase chain reaction; NC, negative control; mRNA, messenger RNA; siRNA, small interfering RNA.

We sought to evaluate the drug response-predictive ability of top identified genes by TINDL, both computationally and experimentally. We focused on tamoxifen due to the good prediction performance of TINDL for this drug (AUROC = 0.92, *P* = 1.14E−3 for Mann–Whitney *U* test). First, using only top implicated genes for this drug (*n* = 136 based on the threshold identified by kneedle), we observed a consistently high value of AUROC and a significant Mann–Whitney *U* test *P* value (Figure 4A, AUROC = 0.89, *P* = 2.32E−3). Next, we reduced the number of genes for the model to only top 20 and observed that AUROC remains high even with this small number of genes (Figure 4A, AUROC = 0.90, *P* = 1.65E−3). This shows that even a small panel of 20 genes can be used to predict the CDR of this drug, suggesting potential clinical applications in precision medicine for these small panels of genes.

Next, we set out to determine whether genes identified by TINDL as predictive of tamoxifen response could be associated *in vitro* with relevant changes in drug sensitivity. We selected 10 genes identified by TINDL, which included the top 9 ranked genes (*RPP25*, *EMP1*, *EXTL3*, *EXOC2*, *NUP37*, *RPL13*, *WBP2NL*, *RPS6*, and *GBP1*) as well as the gene ranked as 19 (*JAK2*), due to its involvement with the type II interferon signaling pathway, an important pathway in cancer [66]. We used estrogen receptor positive breast CCLs, MCF7 and T47D, because tamoxifen has most often been used as the treatment for estrogen receptor positive breast cancer patients in general and 85% of patients in our test dataset for this drug corresponded to breast cancer. We measured the dose–response values of tamoxifen in these two cell lines for these ten genes using CyQUANT assay, which provides an accurate measure of cell numbers based on DNA content [67]. We defined “significance” as a gene knockdown with a significant change in apparent IC50 in comparison with a negative control siRNA. Knockdown of all ten genes with specific siRNAs had a significant effect on tamoxifen sensitivity in MCF7 cell line (*P* < 0.0001, extra sum-of-squares F test), validating 100% of tested genes in this cell line (Figure 4B, Figure S9; Table S7). Similarly, our experiments confirmed seven of these genes in T47D cell line (Figure 4C, Figure S10; Table S7). Taken together, through the functional validation in estrogen receptor positive breast cancer cells, we found that the expression of seven genes, *RPP25*, *EXOC2*, *NUP37*, *RPL13*, *RPS6*, *GBP1*, and *JAK2*, were involved in tamoxifen-induced response in both cell lines, and three genes, *EMP1*, *EXTL3*, and *WBP2NL* were involved in tamoxifen-induced response in MCF7. The percentage of variation in the IC50 of breast cancer cells that was explained by the variation of expression of these ten genes is provided in Table S7, whereas Table S8 shows the efficiency of knockdown for each gene.

## Discussion

Predicting the response of an individual to cancer treatments and identification of predictive biomarkers of drug response are two major goals of individualized medicine. Computational models that can achieve these goals based on preclinical *in vitro* data can make a considerable impact, due to the significant ease of preclinical data generation and data collection compared with clinical samples. This is particularly important for newly developed or newly approved drugs, for which clinical samples may be very limited or non-existent. However, the biological and statistical differences between CCLs and patient tumors make this task challenging. In a recent study [9], we assessed the ability of a wide range of ML models trained on preclinical CCLs, including those that incorporate auxiliary information such as gene interaction networks, in predicting the CDR of cancer patients. Our analysis confirmed the difficulty of this task and emphasized the importance of carefully designing advanced computational techniques.

In this study, we developed TINDL, and showed its substantial improvement compared with the state-of-the-art ML models (based on both traditional and DL techniques) (Figure 1). Our results showed the importance of removing the statistical discrepancies between preclinical and clinical samples, as well as incorporating the cancer types and tissues of origin of the tumor samples. TINDL is not simply a drug response predictor, but rather allows identification of the most predictive biomarkers for each drug. The biomarkers identified by multiple drugs (Figure S8B) suggested important genes and signaling pathways that may play important roles in the mechanism of action of different drugs in cancer. Many genes identified during our study have been reported to have altered levels of expression in response to a given drug, especially *SLFN11* for multiple chemotherapies [42], [43], [44], [68], [69], *SALL4* for cisplatin [70], *ABCB1* for taxane and doxorubicin [71], [72], *PIGB* for gemcitabine [73], and *BAX* to oxaliplatin [74]. These results suggest that our preclinical-to-clinical model could generate biologically relevant candidate genes and pathways for understanding mechanisms underlying drug resistance, and may offer additional combinational therapeutic strategies to overcome certain drug resistance.

Focusing on tamoxifen, we were able to show that only a small panel of 20 genes can preserve the predictive performance of TINDL for this drug (Figure 4A). Moreover, functional validation of 10 of these genes identified by TINDL using siRNA knockdown performed with MCF7 and T47D estrogen receptor positive breast cancer cells, confirmed the direct role of these genes in response to tamoxifen (Figure 4B and C, Figures S9 and S10). These results suggest that, like many complex traits, response to tamoxifen also involves multiple genes in different pathways. In addition, these results provide us with new insights into novel mechanisms in tamoxifen response. For example, among these genes, *RPS6* is the canonical substrate of S6 kinase (S6K), which is activated by integrin engagement and inactivated by detachment. Abnormal expression of *RPS6* has been indicated as a critical trigger for detachment-induced keratinization related to breast cancer development [75]. Indeed, the prognostic value of *RPS6* was assessed by Kaplan–Meier plotter analysis of GEx data from estrogen receptor positive/HER2 negative breast tumor samples of 686 patients. High expression of *RPS6* was associated with better relapse-free survival (RFS) in this cohort of patients (Figure S11A). Decreased phosphorylation of *RPS6* was previously observed in tamoxifen-resistant breast cancer cells compared with parental cells [76]. However, to the best of our knowledge, no previous study has linked *RPS6* to tamoxifen sensitivity. The fact that we found that *RPS6* expression can predict tamoxifen sensitivity and that knockdown of *RPS6* desensitized breast cancer cells to tamoxifen exposure by two folds suggests a potential role for *RPS6* in the estrogen response pathway, in addition to its role as a protein synthesis regulator. In addition to its prognostic value, further analysis revealed that high messenger RNA (mRNA) expression of *RPS6* was also remarkably associated with prolonged RFS in tamoxifen-treated patients (Figure S11B). This hypothesis will need to be tested further in future experiments. The second gene that influenced tamoxifen response the most was *RPL13*, also known as “Ribosomal Protein L13”. *RPL13* encodes a component of the 60S ribosomal subunit that is expressed at significantly higher levels in benign breast lesions than in breast carcinomas [77]. Similarly, to the best of our knowledge, no previous study has linked *RPL13* to estrogen signaling or tamoxifen response. Kaplan–Meier Plotter analysis revealed that patients with high expression of *RPL13* had a significantly longer RFS than those with low *RPL13* expression (Figure S11C). Our observations here suggest an important role of *RPL13* expression level in predicting tamoxifen sensitivity, and could help identify additional drug targets or treatment options to overcome tamoxifen resistance.

Our analysis suggests that TINDL performs better than other approaches (in terms of the number of drugs for which it can distinguish between resistant and sensitive tumors). Although its superior performance compared with traditional ML models can be attributed to higher capacity of DL approaches in modeling complex and nonlinear relationships, its superior performance compared with DL-based domain adaptation techniques reveals its ability to remove the discrepancies between the preclinical and clinical samples. In this study, we performed additional analyses on the embedding space, which confirmed the hypothesis above both visually and quantitatively. When inspecting the principal components and the UMAPs of the samples in the embedding space from the two datasets (Figure 3B, Figures S3–S6), it was clearly visible that the distributions of GDSC and TCGA samples were quite distinct from each other when using domain adaptation models or ComBat. However, embeddings learned by TINDL showed a mixing of the GDSC and TCGA samples, which can be interpreted as a better reduction of the domain discrepancy. We quantified this observation by calculating the average inter-domain distance of the samples in the latent space (smaller value is better). As shown in Figure 3A, TINDL had a significantly lower average distance compared with the existing approaches. One possible reason for this observation is that the other approaches do not incorporate prior information about the target domain. Tissues have distinct GEx profiles, which was leveraged by TINDL. Another reason is the difficulty of assessing the level of adaptation in domain adaptation models because vector representations of GEx (unlike images) cannot be visually verified. Furthermore, domain adaptation methods can suffer from a “mode collapse” problem in which all samples are mapped in a small subspace in the latent space such that the discriminator is confused, which is erroneously equated to having a sufficient adaptation. We would like to point out that in spite of the shortcomings of current domain adaptation techniques, we posit that novel domain adaptation methods can be developed to improve the results. However, such methods need to be carefully designed for the analysis of GEx data and must take into account biological factors that influence the response of cancer patients to different drugs. In addition, including information on the cancer type or even subtype of each cancer may be necessary to achieve better results.

Another important consideration is that due to the limitation of CCLs in mimicking patient tumors (*e.g.*, their growth in 2D environment, being more homogenous than tumors, and not being able to capture the effect of tumor microenvironment), computational models trained on CCLs are limited in their ability to predict CDR of cancer patients, even if they remove the statistical discrepancies of the training and test sets. As a result, availability of large datasets, pertaining to better models of cancer (such as patient-derived organoids or xenografts), plays an important role in improving the predictive ability of computational models.

In this study, our focus was models trained only on GEx profiles of samples, because previous studies have shown this data modality to be most informative regarding drug response [6]. However, a multi-omics approach that incorporates different molecular characteristics of samples may provide a more complete understanding of the mechanisms of drug response in cancer. Nevertheless, such models need to be carefully designed to avoid over-fitting due to the additional number of features, which can cause severe performance deterioration. Another limitation of this study was that all the computational models were trained on CCLs and their response to single drugs. However, some of the patients in the TCGA dataset have received multiple drugs in the course of their treatment, which we had to include in the analysis due to the small number of samples with known CDR. In such cases, any computational models trained on single drugs can only provide an approximation. To improve the prediction performance in such cases, a computational model must also consider the synergistic and antagonistic effects of the drugs. Recent large publicly available datasets such as DrugComb [78] and DrugCombDB [79] that contain response of different cell lines to pairs of drugs provide an opportunity for developing such methods, a direction that we will pursue in the future.

## Materials and methods

### Datasets

We used the publicly available data from GDSC and TCGA for training and testing, respectively. For training data, we used the robust multi-array analysis (RMA)-normalized GEx data in GDSC, which contains 958 unique cell lines. For the test data, we used RNA sequencing [in fragments per kilobase million (FPKM)] from primary tumors in TCGA. For both datasets, we filtered out genes with missing values. We also removed genes that were not expressed (FPKM < 1) for at least 90% of all the TCGA samples, and transformed the remaining genes using log_2_ (FPKM + 0.1). Only genes that were present in both datasets were included, which summed up to 15,650 genes. We used z-score to normalize the GDSC GEx data (gene-wise) as well as the ln IC50 values (drug-wise). We obtained CDR of cancer patients from the supplementary file of Ding and his colleagues [10]. Because the number of samples with known drug response in TCGA is relatively small, in our analysis we also included samples that have received multiple drugs in their course of treatment. We only focused on drugs which are common to both datasets and have at least 20 samples with known CDR in TCGA. We used a tissue-informed normalization, which is detailed below. Furthermore, we recategorized the CDRs to sensitive (corresponding to complete and partial responses) and resistant (corresponding to stable disease and clinically progressive disease). Details on sample counts and tissue types per drug are in Table S1.

### Tissue-informed normalization

TINDL trained a separate model for each drug. Each model performed a separate normalization on the GEx profiles of test samples from TCGA to account for the cancer types and tissues of origin of the samples. First, for each drug D, the set of tissues/cancer types to which this drug was administered in the TCGA samples was identified (referred to as $T_{D}$). All samples corresponding to $T_{D}$ (excluding those used in the test set) were collected from TCGA, forming the unlabeled dataset. Then, the gene-wise mean ($\mu_{T_{D}}$) and standard deviation ($\sigma_{T_{D}}$) of these unlabeled samples were calculated and used to normalize labeled test samples corresponding to drug D. More specifically, for a gene i of an arbitrary sample in the test set, the normalized value $x_{i}$ would be:

(1) $$x_{i}=\frac{{\overset{∼}{x}}_{i}-\mu_{i,T_{D}}}{\sigma_{i,T_{D}}}$$

where  ${\tilde{x}}_{i}$ is the log-transformed expression for gene i of that sample. The test samples were then used as input to the trained model to predict the normalized ln IC50 values, which were compared with the actual CDR categories for evaluation.

### Architecture of TINDL, hyperparameter selection, and training

We used grid-search and 5-fold cross validation to select the number of epochs, batch size, and learning rate of all our DL-based models (including TINDL). We only used the training data corresponding to CCLs (from GDSC) to perform the hyperparameter search, in which the set of hyperparameters with the highest average Pearson correlation coefficient on the validation set across the five folds were chosen. Specific hyperparameters chosen using this procedure for TINDL are provided in Table S9. In addition to the input layer (which contained one node for each gene), we used three hidden layers with dense connections, each with 512, 256, and 128 hidden nodes, in the order of their distance to the input layer. We used a rectified linear units (ReLU) activation function and added a dropout layer with 0.2 probability of dropping out prior to the output layer.

Models were trained using mean squared error (MSE) as the loss function, and the normalized ln IC50 values as the labels. During hyperparameter tuning, models were allowed to train up to a maximum of 1000 epochs, but early stopping was applied when the loss of model did not decrease after 30 epochs. After hyperparameter tuning, we retrained a final model using all the labeled CCL samples. We used 10 different random initializations (*i.e.*, seeds) and ensembled the models by averaging their predictions to ensure robustness of the results. Note that individual models were trained independently. Loss curves for hyperparameter tuning and final training are shown in Figures S12 and S13. A similar technique was used for ADDA-DL, DANN-DL, ComBat-DL, TrainNorm-DL, and TestNorm-DL.

### Calculation of contribution scores of genes

In the second phase of TINDL (Figure 1B), we used CXPlain [18] as the explainer to assign a contribution score to each gene in each sample. CXPlain is a method that attempts to provide causal explanations of predictions of a trained model. This is achieved by training a separate model (called “explainer”) using the outputs of the trained model (called “predictor”). This method utilizes Granger causality [80] to evaluate the contribution of a single feature (gene in our case) by zeroing out features one by one and calculating the normalized difference of the predictor’s original error and its error when the feature is zeroed out. In our case, we defined error as $\varepsilon_{X}={\left(y_{X}-{\hat{y}}_{X}\right)}^{2}$, where $y_{X}$ is the true value and ${\hat{y}}_{X}$ is the output of the predictor for sample $X=\left\{x_{1},\cdots ,x_{p}\right\}$, p being the number of features. Note that our predictor was an ensemble, and ${\hat{y}}_{X}$ is the average of the outputs of the individual models. Prior to training the explainer, the real contribution vectors, $\Omega_{X}=\left\{\omega_{1}\left(X\right),\cdots ,\omega_{p}\left(X\right)\right\}$, are calculated for each training sample as follows:

(2) $$\omega_{i}\left(X\right)=\frac{\Delta \varepsilon_{X,i}}{\sum_{j=1\cdots p}\Delta \varepsilon_{X,j}}$$

where $\Delta \varepsilon_{X,i}=\varepsilon_{X\backslash \{i\}}-\varepsilon_{X}$. Here, $\varepsilon_{X\backslash \{i\}}$ denotes the predictor’s error when given X but with feature i zeroed out. The explainer has an architecture such that the dimensions of the input vector X and the output vector ${\hat{\Omega }}_{X}=\left\{{\hat{\omega }}_{1}\left(X\right),{\cdots ,\hat{\omega }}_{p}\left(X\right)\right\}$ are the same. Each of the outputs correspond to the predicted contribution for the corresponding feature. The explainer is trained by minimizing the Kullback–Leibler (KL) divergence $\mathrm{KL}(\Omega_{X},{\hat{\Omega }}_{X})$ of the real contributions $\Omega_{X}$ and predicted contributions ${\hat{\Omega }}_{X}$ of the training set.

We used a neural network with two layers and 512 hidden units for the explainer, and used the ensemble mode, which trained 10 independent explainers and reported their median as the final contribution values. We modified the code of CXPlain library to fit our application, which we also included in our published code. Once trained, we predicted the contribution values of each genes in each of the samples in the testing set. To obtain drug-specific gene contribution scores, we calculated the mean contribution score of each gene across all the labeled test samples for that drug and normalized it such that the largest contribution score of a drug equals 1.

### Identification of genes with highest contribution scores

After obtaining contribution scores to each gene for a drug, we sought to identify the top genes that substantially affect the predictions our model. We sorted the genes according to their final test contribution scores and plotted a curve (Figure S7), where the X-axis is the rank of the gene i and the Y-axis is the drug-specific contribution score  ${\overset{¯}{\omega }}_{i}$ of gene i. We used the kneedle algorithm [24] to identify the point of maximum curvature, called “knee”, which we then treated as the cutoff for the top genes. Kneedle relies on the idea that if one forms a line l from (1, ${\overset{¯}{\omega }}_{max}$) to ($n,{\overset{¯}{\omega }}_{min}$) and rotate the curve around the point ($n,{\overset{¯}{\omega }}_{min}$), the “knee” can be approximated by the set of points in the local maxima. Among these points, the point that is farthest from the line l is then identified as the knee.

### Knowledge-guided pathway enrichment analysis

We identified pathways associated with the top identified genes using KnowEng’s GSC pipeline [47]. We used the network-guided mode, which incorporates knowledge in the form of gene–gene interactions to augment the analysis. For the knowledge network, we selected the experimentally verified protein–protein interactions within the STRING database [81]. We then proceeded with the default 50% network smoothing parameter and used the “Enrichr” pathway collection. This pipeline does not provide a *P* value, but rather uses a score called “Difference Score” to implicate top pathways. Any pathway above the 0.5 threshold is considered associated with the input query set. A value above this threshold shows that the pathway has a high relevance score to the input query set (using a random walk with restarts algorithm), compared with the background [47].

### Precision at *k*-th percentile

For each drug, we used TINDL’s predictions of ln IC50 of the tumor samples, and identified the *k*-th percentiles of the distribution (*k* ≤ 50), which we denoted as $t_{k}$. We stratified the predictions such that all predictions below $t_{k}$ was predicted as positives (*i.e.*, sensitive). We then calculated the precision at *k*-th percentile as $\frac{TP_{k}}{TP_{k}+FP_{k}}$, where $TP_{k}$ and $FP_{k}$ are the true positives and false positives at *k*-th percentile, respectively.

### Baseline models

SVR, random forests, and LASSO regression were all implemented using scikit-learn. Geeleher’s method [14] was reimplemented using scikit-learn and pyComBat, a python implementation of ComBat [15]. We used the available implementation of TG-LASSO [9]. All hyperparameters were tuned as described in the previous subsections except for TG-LASSO, which has its built-in hyperparameter tuning.

To ensure a fair comparison, all DL-based baseline models used a similar architecture to TINDL. Additionally, the hyperparameter tuning and training procedure was also similar to the one described above for TINDL. Below, we describe model-specific considerations. For ComBat-DL we used ComBat [15] for removing the discrepancy between TCGA and GDSC datasets. Similar to TINDL, we used both labeled and unlabeled samples of TCGA for this purpose.

ADDA-DL utilizes ADDA [17], to remove the discrepancy between TCGA and GDSC datasets. ADDA is a unidirectional domain adaptation technique, which takes a pretrained neural network and attempts to adapt the network to the target dataset by forcing the latent feature space of the target dataset (TCGA) to be similar to that of the source dataset (GDSC). We used the TINDL model as the pretrained network, which we adapt through the adversarial losses of ADDA. We used the unlabeled tumor samples from the drugs target tissues during training. Details are provided in File S1.

DANN-DL utilizes DANN [16] to remove the discrepancy between TCGA and GDSC datasets. DANN utilizes the shared latent feature space to allow the model to be used on the target dataset despite only being trained using the labels of source dataset. This is done by incorporating a gradient-reversed discriminative loss function such that a discriminator cannot tell whether the given embedding came from the source (GDSC) or target (TCGA) datasets. Similar to ADDA-DL, we used the unlabeled tumor samples from the drugs target tissues for training of the discriminator.

TrainNorm-DL and TestNorm-DL are two default workflows when domain discrepancies are not an important problem. In the TrainNorm-DL, we used the training set’s mean and standard deviation to normalize both the training set and the test set. This is analogous to assuming that the training set and test set belong to the same domain. The TestNorm-DL uses a per dataset normalization technique, in which the test set is normalized using its own mean and standard deviation, whereas the training set also uses its own summary statistics. The same model as TINDL was used for these baselines because the difference in normalization only affects the test set.

GCN [22] and GAT [23] are two types of graph neural networks. For both architectures, the STRING co-expression graph [81] was used as the input structure. Only genes that existed in both STRING and the transcriptomic dataset were utilized. Each node in the graph is a gene, represented by the concatenation of a unique trainable embedding vector (gene-specific, shared across samples) and the expression value of gene (sample-specific). The purpose of gene-specific vectors is to allow GCN and GAT to distinguish differences between genes, which would normally be ignored because of the permutation invariance properties of architectures. The complete model is similar to that of TINDL, but with the first two layers replaced with GCN or GAT, corresponding to two-hop message passing in the graph.

LSTM is a type of recurrent neural network, which are typically used for sequential data. We used the gene indices of our input file as the artificially induced ordering, and split the features into ten windows. Only the embedding coming from the last window (10th pass to the LSTM) was fed to the subsequent fully-connected layers. Only one LSTM layer was used because the parameters of one layer of LSTM are more comparable to two layers of a fully-connected network. The complete model resembles TINDL, but with the first layer replaced with an LSTM layer.

### Measurement of distance of clinical and preclinical samples in the latent space of DL-based models

To assess the ability of each DL-based model in removing discrepancy between preclinical and clinical samples, we used pairwise Euclidean distance of samples based on their representation learned by the encoder of the DL models. Because these representations are used by the decoder to make predictions, comparing these latent representations is more meaningful than comparing input feature representations. We used Ward’s method [82] to assess the distance of preclinical samples and clinical samples, which is one of the most popular methods in assessing the distance of two groups of samples. This method, which is widely used in hierarchical clustering, has the advantage that not only analyzes the Euclidean distances of the data points, but also incorporates their variance in determining the distance of two groups of samples.

### Chemicals and reagents

Dulbecco’s Modified Eagle’s medium (DMEM; Catalog No. 11-965-092) was purchased from ThermoFisher Scientific (Carlsbad, CA). Fetal bovine serum (FBS; Catalog No. 10-437-028) and charcoal-stripped FBS (Catalog No. 12-676-029) were from Invitrogen (Carlsbad, CA). On-Target Plus SMARTpool siRNAs targeting *RPP25*, *EMP1*, *EXTL3*, *EXOC2*, *NUP37*, *RPL13*, *WBP2NL*, *RPS6*, *GBP1*, and *JAK2* as well as negative siRNA controls were purchased from Dharmacon (Horizon Discovery, Lafayette, CO). Reagents and primers for quantitative real-time polymerase chain reaction (qRT-PCR) were purchased from QIAGEN (Valencia, CA) and Integrated DNA Technologies (Coralville, IA). 17β-estradiol (E2; Catalog No. E2758) and 4-hydroxytamoxifen (OH-TAM; Catalog No. 579002) were purchased from Sigma Aldrich (Saint Louis, MO).

### Cell lines

MCF7 and T47D cell lines were obtained from American Type Culture Collection (ATCC; Manassus, VA) in 2014, and the identities of all cell lines were confirmed by the medical genome facility at Mayo Clinic (Rochester, MN) using short tandem repeat profiling upon receipt. MCF7 cells were cultured in DMEM containing 10% FBS. T47D cells were cultured in RPMI-1640 containing 10% FBS.

### Transfection and gene silencing

Specific siRNAs that targeted *RPP25*, *EMP1*, *EXTL3*, *EXOC2*, *NUP37*, *RPL13*, *WBP2NL*, *RPS6*, *GBP1*, *JAK2*, and negative siRNA controls (Horizon Discovery) were transfected into MCF7 and T47D cells in 96-well plates using Lipofectamine RNAiMAX Transfection Reagent (Catalog No. 13778500, ThermoFisher Scientific, Waltham, MA) according to the vendor’s protocol [67], [83]. Total RNA was extracted 48 h after transfection for RNA quantification. Specific siGENOME siRNA SMARTpool Reagents (Catalog Nos. M-020782-01-0005 for *RPP25*, M-010507-00-0005 for *EMP1*, M-012578-00-0005 for *EXTL3*, M-017357-01-0005 for *EXOC2*, M-014282-00-0005 for *NUP37*, M-013714-00-0005 for *RPL13*, M-017184-00-0005 for *WBP2NL*, M-003024-01-0005 for *RPS6*, M-005153-02-0005 for *GBP1*, and M-003146-02-0005 for *JAK2*) against a given gene as well as a negative control, siGENOME Non-Targeting siRNA (Catalog No. D-001206-13-20), were purchased from Horizon Discovery. For the purpose of drug tamoxifen response assay, cells were plated in base medium supplemented with 5% charcoal stripped FBS for 24 h, and then cultured in FBS-free DMEM media for another 24 h before transfection. Different treatments were started 24 h after transfection.

### qRT-PCR

qRT-PCR assays were performed for measuring GEx using Power SYBR Green RNA-to-CT 1-Step Kit (Catalog No. 4389986, ThermoFisher Scientific, Grand Island, NY) and PrimeTime (Integrated DNA Technologies, Coralville, IA) pre-designed quantitative polymerase chain reaction (qPCR) primers. RNA was extracted using the QIAGEN RNeasy Kit (Catalog No. 74104, QIAGEN, Germantown, MD). RNA was measured by NanoDrops3000 (ThermoFisher Scientific, Rockford, IL). qRT-PCR reactions were prepared as per the manufacturer’s protocol. Samples were run using StepOnePlus Real-Time PCR System (ThermoFisher Scientific, Carlsbad, CA). For the experiments, we used three technical replicates. GEx was normalized to the negative siRNA control. Table S8 shows the knockdown efficiency of each gene and corresponding statistical analysis.

### Tamoxifen sensitivity assay

Drugs were dissolved in dimethyl sulfoxide (DMSO), and aliquots of stock solutions were frozen at −80 °C. Cytotoxicity assays were performed in triplicate at each drug concentration. Specifically, 4000 breast cancer cells were seeded in 96-well plates, cultured in base media containing 5% (v/v) charcoal-stripped FBS for 24 h, and subsequently cultured in FBS-free base media for another 24 h. Cells were then transfected with either control siRNA or siRNA targeting a specific gene. After 24-h transfection, the media were replaced with fresh FBS-free base media, and the cells were treated with 10 μl of tamoxifen at final concentrations of 0, 0.1875, 0.375, 0.75, 1.5, 3, 6, 12, 24, and 48 μM [84]. After incubation for an additional 72 h, cytotoxicity was determined by quantification of DNA content using CyQUANT assay (Catalog No. C35012, Invitrogen, Carlsbad, CA) following the manufacturer’s instructions [85], [86], [87]. 100 μl of CyQUANT assay solution was added, and plates were incubated at 37 °C for 1 h and then read in a Safire2 Microplate Reader with filters appropriate for 480-nm excitation and 520-nm emission.

## Code availability

An implementation of TINDL in Python, with appropriate documentation, is available at https://github.com/ddhostallero/tindl. Preprocessed input data and trained models are also linked in the code repository.

## Competing interests

The authors have declared no competing interests.

## CRediT authorship contribution statement

**David Earl Hostallero:** Methodology, Software, Formal analysis, Visualization, Data curation, Writing – original draft, Writing – review & editing. **Lixuan Wei:** Investigation, Visualization, Writing – review & editing. **Liewei Wang:** Investigation, Writing – review & editing. **Junmei Cairns:** Conceptualization, Supervision, Funding acquisition, Writing – review & editing. **Amin Emad:** Conceptualization, Methodology, Formal analysis, Supervision, Funding acquisition, Writing – original draft, Writing – review & editing. All authors have read and approved the final manuscript.

## Supplementary material

Supplementary File S1 **Supplementary methods**

Supplementary Figure S1 **ROC curves of TINDL and baseline approaches in different drugs** LASSO, least absolute shrinkage and selection operator; SVR, support vector regression; RF, random forest; TG-LASSO, tissue guided LASSO

Supplementary Figure S2 **Precision–recall curves of TINDL and baseline approaches in different drugs**

Supplementary Figure S3 **UMAP and PCA plots of the learned latent features of different drugs by TINDL** Purple points indicate cell line samples (GDSC) and orange points indicate tumor samples (TCGA). UMAP, Uniform Manifold Approximation and Projection.

Supplementary Figure S4 **UMAP and PCA plots of the learned latent features of different drugs by Combat-DL** Purple points indicate cell line samples (GDSC) and orange points indicate tumor samples (TCGA).

Supplementary Figure S5 **UMAP and PCA plots of the learned latent features of different drugs by ADDA-DL** Purple points indicate cell line samples (GDSC) and orange points indicate tumor samples (TCGA).

Supplementary Figure S6 **UMAP and PCA plots of the learned latent features of different drugs by DANN-DL** Purple points indicate cell line samples (GDSC) and orange points indicate tumor samples (TCGA).

Supplementary Figure S7 **Contribution scores of genes in the trained model of TINDL** The Y-axis shows the contribution score, and the X-axis shows genes in a descending order of their score. Orange points indicate the “knees” found by kneedle algorithm, representing the threshold below which the contribution of genes to the trained model is small. The numbers in the brackets show number of genes above threshold and threshold.

Supplementary Figure S8 **Genes identified by TINDL for different drugs A.** Heatmap of the Jaccard similarity of the selected top genes in the 14 drugs. **B.** Number of drugs in which the genes were identified as a top gene. Only genes that were present in the top genes of at least four drugs are included.

Supplementary Figure S9 **Tamoxifen dose–response curves corresponding to the siRNA knockdown of 10 genes identified by TINDL in MCF7 cells** Knockdown efficiency was assessed by qRT-PCR using three technical replicates (Table S8). Gene expression was normalized to siRNA negative control. Table S8 shows the knockdown efficiency of each gene and corresponding statistical analysis.

Supplementary Figure S10 **Tamoxifen dose–response curves corresponding to the siRNA knockdown of 10 genes identified by TINDL in T47D cells** Knockdown efficiency was assessed by qRT-PCR using three technical replicates (Table S8). Gene expression was normalized to siRNA negative control. Table S8 shows the knockdown efficiency of each gene and corresponding statistical analysis.

Supplementary Figure S11 **Kaplan–Meier survival analysis of *RPS6* and *RPL13* gene expression in estrogen receptor positive/HER2 negative breast cancer patients using Kaplan–Meier Plotter A.** RFS of *RPS6* in systemically untreated patients. **B.** RFS of *RPS6* in tamoxifen treated patients. **C.** RFS of *RPL13* in tamoxifen treated patients. RFS, relapse free survival; HR, hazard ratio.

Supplementary Figure S12 **MSE curves of different hyperparameters during grid-search** To reduce cluttering, only the average value across the five folds were plotted. The shaded regions are the 90% confidence intervals. The chosen number of epochs is denoted as a vertical line, while the purple curves represent the training and validation curves of the chosen set of hyperparameters. MSE, mean squared error.

Supplementary Figure S13 **Training MSE curves of the 10 different initializations during the final training** Individual models are denoted as the gray curves while their average is denoted by the purple curve. The final training uses all the labeled cell lines as the training set so there are no validation curves.

Supplementary Table S1 **Information regarding the samples used in both training and testing sets**

Supplementary Table S2 **Performance of different models in predicting CDR**

Supplementary Table S3 **Precision at k^th^ percentile of TINDL**

Supplementary Table S4 **List of top genes identified by our pipeline**

Supplementary Table S5 **Genes and pathways associated with multiple drugs**

Supplementary Table S6 **Pathways associated with genes implicated for each drug by TINDL**

Supplementary Table S7 **The result of siRNA gene knockdown experiments in MCF7 and T47D cell lines for 10 genes identified by TINDL for tamoxifen**

Supplementary Table S8 **Efficiency of knockdown experiments**

Supplementary Table S9 **Learning rates, and batch sizes, and number of epochs used in the final models**

## References

[1] Sung H., Ferlay J., Siegel R.L., Laversanne M., Soerjomataram I., Jemal A. (2021). Global Cancer Statistics 2020: GLOBOCAN estimates of incidence and mortality worldwide for 36 cancers in 185 countries. CA Cancer J Clin.

[2] Cancer Genome Atlas Research Network, Weinstein J.N., Collisson E.A., Mills G.B., Shaw K.R., Ozenberger B.A. (2013). The Cancer Genome Atlas Pan-Cancer analysis project. Nat Genet.

[3] Yang W., Soares J., Greninger P., Edelman E.J., Lightfoot H., Forbes S. (2013). Genomics of Drug Sensitivity in Cancer (GDSC): a resource for therapeutic biomarker discovery in cancer cells. Nucleic Acids Res.

[4] Barretina J., Caponigro G., Stransky N., Venkatesan K., Margolin A.A., Kim S. (2012). The Cancer Cell Line Encyclopedia enables predictive modelling of anticancer drug sensitivity. Nature.

[5] Basu A., Bodycombe N.E., Cheah J.H., Price E.V., Liu K., Schaefer G.I. (2013). An interactive resource to identify cancer genetic and lineage dependencies targeted by small molecules. Cell.

[6] Costello J.C., Heiser L.M., Georgii E., Gonen M., Menden M.P., Wang N.J. (2014). A community effort to assess and improve drug sensitivity prediction algorithms. Nat Biotechnol.

[7] Jiang P., Sellers W.R., Liu X.S. (2018). Big data approaches for modeling response and resistance to cancer drugs. Annu Rev Biomed Data Sci.

[8] Yang J., Li A., Li Y., Guo X., Wang M. (2019). A novel approach for drug response prediction in cancer cell lines via network representation learning. Bioinformatics.

[9] Huang E.W., Bhope A., Lim J., Sinha S., Emad A. (2020). Tissue-guided LASSO for prediction of clinical drug response using preclinical samples. PLoS Comput Biol.

[10] Ding Z., Zu S., Gu J. (2016). Evaluating the molecule-based prediction of clinical drug responses in cancer. Bioinformatics.

[11] Wang Z., Li R., Wang M., Li A. (2021). GPDBN: deep bilinear network integrating both genomic data and pathological images for breast cancer prognosis prediction. Bioinformatics.

[12] Malik V., Kalakoti Y., Sundar D. (2021). Deep learning assisted multi-omics integration for survival and drug-response prediction in breast cancer. BMC Genomics.

[13] Sharifi-Noghabi H., Peng S., Zolotareva O., Collins C.C., Ester M. (2020). AITL: adversarial inductive transfer learning with input and output space adaptation for pharmacogenomics. Bioinformatics.

[14] Geeleher P., Cox N.J., Huang R.S. (2014). Clinical drug response can be predicted using baseline gene expression levels and *in vitro* drug sensitivity in cell lines. Genome Biol.

[15] Johnson W.E., Li C., Rabinovic A. (2007). Adjusting batch effects in microarray expression data using empirical Bayes methods. Biostatistics.

[16] Ganin Y, Lempitsky V. Unsupervised domain adaptation by backpropagation. Proc 32nd Int Conf Mach Learn 2015:1180–9.

[17] Tzeng E., Hoffman J., Saenko K., Darrell T. (2017). Adversarial Discriminative Domain Adaptation. IEEE Conf Comput Vis Pattern Recognit.

[18] Schwab P, Karlen W. CXPlain: causal explanations for model interpretation under uncertainty. Proc 33rd Int Conf Neural Inf Process Syst 2019:10220–30.

[19] Dong Z., Zhang N., Li C., Wang H., Fang Y., Wang J. (2015). Anticancer drug sensitivity prediction in cell lines from baseline gene expression through recursive feature selection. BMC Cancer.

[20] Li B., Severson E., Pignon J.C., Zhao H., Li T., Novak J. (2016). Comprehensive analyses of tumor immunity: implications for cancer immunotherapy. Genome Biol.

[21] Butler A., Hoffman P., Smibert P., Papalexi E., Satija R. (2018). Integrating single-cell transcriptomic data across different conditions, technologies, and species. Nat Biotechnol.

[22] Kipf TN, Welling M. Semi-supervised classification with graph convolutional networks. Int Conf Learn Represent 2017.

[23] Velickovic P, Cucurull G, Casanova A, Romero A, Liò P, Bengio Y. Graph attention networks. Int Conf Learn Represent 2018.

[24] Satopaa V, Albrecht J, Irwin D, Raghavan B. Finding a “kneedle” in a haystack: detecting knee points in system behavior. 31st Int Conf Distrib Comput Syst Workshop 2011:166–71.

[25] Liu M.L., Zang F., Zhang S.J. (2019). RBCK1 contributes to chemoresistance and stemness in colorectal cancer (CRC). Biomed Pharmacother.

[26] Chen T.J., Chou C.L., Tian Y.F., Yeh C.F., Chan T.C., He H.L. (2021). High FRMD3 expression is prognostic for worse survival in rectal cancer patients treated with CCRT. Int J Clin Oncol.

[27] Kim E.J., Kim S.H., Jin X., Jin X., Kim H. (2017). KCTD2, an adaptor of Cullin3 E3 ubiquitin ligase, suppresses gliomagenesis by destabilizing c-Myc. Cell Death Differ.

[28] Longatto-Filho A., Fregnani J.H., da Costa A.M., de Araujo-Souza P.S., Scapulatempo-Neto C., Herbster S. (2021). Evaluation of elafin immunohistochemical expression as marker of cervical cancer severity. Acta Cytol.

[29] Li L.Y., Yang Q., Jiang Y.Y., Yang W., Jiang Y., Li X. (2021). Interplay and cooperation between SREBF1 and master transcription factors regulate lipid metabolism and tumor-promoting pathways in squamous cancer. Nat Commun.

[30] Deng J., Chen X., Zhan T., Chen M., Yan X., Huang X. (2021). CRYAB predicts clinical prognosis and is associated with immunocyte infiltration in colorectal cancer. PeerJ.

[31] Fredriksson R., Sreedharan S., Nordenankar K., Alsio J., Lindberg F.A., Hutchinson A. (2019). The polyamine transporter Slc18b1(VPAT) is important for both short and long time memory and for regulation of polyamine content in the brain. PLoS Genet.

[32] Liu Y., Li Q., Zhou L., Xie N., Nice E.C., Zhang H. (2016). Cancer drug resistance: redox resetting renders a way. Oncotarget.

[33] Abdallah H.M., Al-Abd A.M., El-Dine R.S., El-Halawany A.M. (2015). P-glycoprotein inhibitors of natural origin as potential tumor chemo-sensitizers: a review. J Adv Res.

[34] Chen K.G., Valencia J.C., Gillet J.P., Hearing V.J., Gottesman M.M. (2009). Involvement of ABC transporters in melanogenesis and the development of multidrug resistance of melanoma. Pigment Cell Melanoma Res.

[35] Barzak F.M., Harjes S., Kvach M.V., Kurup H.M., Jameson G.B., Filichev V.V. (2019). Selective inhibition of APOBEC3 enzymes by single-stranded DNAs containing 2′-deoxyzebularine. Org Biomol Chem.

[36] Liu F., Wei J., Hao Y., Lan J., Li W., Weng J. (2021). Long intergenic non-protein coding RNA 02570 promotes nasopharyngeal carcinoma progression by adsorbing microRNA miR-4649-3p thereby upregulating both sterol regulatory element binding protein 1, and fatty acid synthase. Bioengineered.

[37] Hu G., Zhang J., Xu F., Deng H., Zhang W., Kang S. (2018). Stomatin-like protein 2 inhibits cisplatin-induced apoptosis through MEK/ERK signaling and the mitochondrial apoptosis pathway in cervical cancer cells. Cancer Sci.

[38] Green A.M., Budagyan K., Hayer K.E., Reed M.A., Savani M.R., Wertheim G.B. (2017). Cytosine deaminase APOBEC3A sensitizes leukemia cells to inhibition of the DNA replication checkpoint. Cancer Res.

[39] Petljak M., Dananberg A., Chu K., Bergstrom E.N., Striepen J., von Morgen P. (2022). Mechanisms of APOBEC3 mutagenesis in human cancer cells. Nature.

[40] Burrell R.A., McClelland S.E., Endesfelder D., Groth P., Weller M.C., Shaikh N. (2013). Replication stress links structural and numerical cancer chromosomal instability. Nature.

[41] Hanahan D., Weinberg R.A. (2011). Hallmarks of cancer: the next generation. Cell.

[42] Murai J., Thomas A., Miettinen M., Pommier Y. (2019). Schlafen 11 (SLFN11), a restriction factor for replicative stress induced by DNA-targeting anti-cancer therapies. Pharmacol Ther.

[43] Deng Y., Cai Y., Huang Y., Yang Z., Bai Y., Liu Y. (2015). High SLFN11 expression predicts better survival for patients with KRAS exon 2 wild type colorectal cancer after treated with adjuvant oxaliplatin-based treatment. BMC Cancer.

[44] Winkler C., Armenia J., Jones G.N., Tobalina L., Sale M.J., Petreus T. (2021). SLFN11 informs on standard of care and novel treatments in a wide range of cancer models. Br J Cancer.

[45] Gardner E.E., Lok B.H., Schneeberger V.E., Desmeules P., Miles L.A., Arnold P.K. (2017). Chemosensitive relapse in small cell lung cancer proceeds through an EZH2–SLFN11 axis. Cancer Cell.

[46] Fillmore C.M., Xu C., Desai P.T., Berry J.M., Rowbotham S.P., Lin Y.J. (2015). EZH2 inhibition sensitizes *BRG1* and *EGFR* mutant lung tumours to TopoII inhibitors. Nature.

[47] Blatti C., Emad A., Berry M.J., Gatzke L., Epstein M., Lanier D. (2020). Knowledge-guided analysis of “omics” data using the KnowEnG cloud platform. PLoS Biol.

[48] Gelman A.E., Zhang J., Choi Y., Turka L.A. (2004). Toll-like receptor ligands directly promote activated CD4^+^ T cell survival. J Immunol.

[49] Alexopoulou L., Holt A.C., Medzhitov R., Flavell R.A. (2001). Recognition of double-stranded RNA and activation of NF-κB by Toll-like receptor 3. Nature.

[50] Li J.K., Balic J.J., Yu L., Jenkins B. (2017). TLR agonists as adjuvants for cancer vaccines. Adv Exp Med Biol.

[51] Nowak A.K., Robinson B.W., Lake R.A. (2003). Synergy between chemotherapy and immunotherapy in the treatment of established murine solid tumors. Cancer Res.

[52] Rakoff-Nahoum S., Medzhitov R. (2009). Toll-like receptors and cancer. Nat Rev Cancer.

[53] Kawashima M., Suzuki S.O., Doh-ura K., Iwaki T. (2000). α-Synuclein is expressed in a variety of brain tumors showing neuronal differentiation. Acta Neuropathol.

[54] Ge Y., Xu K. (2016). Alpha-synuclein contributes to malignant progression of human meningioma via the Akt/mTOR pathway. Cancer Cell Int.

[55] Shekoohi S., Rajasekaran S., Patel D., Yang S., Liu W., Huang S. (2021). Knocking out α-synuclein in melanoma cells dysregulates cellular iron metabolism and suppresses tumor growth. Sci Rep.

[56] Tzivion G., Luo Z., Avruch J. (1998). A dimeric 14-3-3 protein is an essential cofactor for Raf kinase activity. Nature.

[57] Clancy J.W., Zhang Y., Sheehan C., D’Souza-Schorey C. (2019). An ARF6–Exportin-5 axis delivers pre-miRNA cargo to tumour microvesicles. Nat Cell Biol.

[58] Clancy J.W., Tricarico C.J., Marous D.R., D’Souza-Schorey C. (2019). Coordinated regulation of intracellular fascin distribution governs tumor microvesicle release and invasive cell capacity. Mol Cell Biol.

[59] Li R., Peng C., Zhang X., Wu Y., Pan S., Xiao Y. (2017). Roles of Arf6 in cancer cell invasion, metastasis and proliferation. Life Sci.

[60] Hu Z., Xu R., Liu J., Zhang Y., Du J., Li W. (2013). GEP100 regulates epidermal growth factor-induced MDA-MB-231 breast cancer cell invasion through the activation of Arf6/ERK/uPAR signaling pathway. Exp Cell Res.

[61] Hopkins B.D., Pauli C., Du X., Wang D.G., Li X., Wu D. (2018). Suppression of insulin feedback enhances the efficacy of PI3K inhibitors. Nature.

[62] Hua H., Kong Q., Yin J., Zhang J., Jiang Y. (2020). Insulin-like growth factor receptor signaling in tumorigenesis and drug resistance: a challenge for cancer therapy. J Hematol Oncol.

[63] Agrawal S., Wozniak M., Luc M., Makuch S., Pielka E., Agrawal A.K. (2019). Insulin enhancement of the antitumor activity of chemotherapeutic agents in colorectal cancer is linked with downregulating PIK3CA and GRB2. Sci Rep.

[64] Bodemann B.O., White M.A. (2008). Ral GTPases and cancer: linchpin support of the tumorigenic platform. Nat Rev Cancer.

[65] Neel N.F., Martin T.D., Stratford J.K., Zand T.P., Reiner D.J., Der C.J. (2011). The RalGEF–Ral effector signaling network: the road less traveled for anti-Ras drug discovery. Genes Cancer.

[66] Gocher A.M., Workman C.J., Vignali D.A.A. (2022). Interferon-γ: teammate or opponent in the tumour microenvironment?. Nat Rev Immunol.

[67] Cairns J., Ly R.C., Niu N., Kalari K.R., Carlson E.E., Wang L. (2020). CDC25B partners with PP2A to induce AMPK activation and tumor suppression in triple negative breast cancer. NAR. Cancer.

[68] Coleman N., Zhang B., Byers L.A., Yap T.A. (2021). The role of Schlafen 11 (SLFN11) as a predictive biomarker for targeting the DNA damage response. Br J Cancer.

[69] Luan J., Gao X., Hu F., Zhang Y., Gou X. (2020). SLFN11 is a general target for enhancing the sensitivity of cancer to chemotherapy (DNA-damaging agents). J Drug Target.

[70] Li Y., Wang M., Yang M., Xiao Y., Jian Y., Shi D. (2021). Nicotine-induced ILF2 facilitates nuclear mRNA export of pluripotency factors to promote stemness and chemoresistance in human esophageal cancer. Cancer Res.

[71] Kikuchi H., Maishi N., Annan D.A., Alam M.T., Dawood R.I.H., Sato M. (2020). Chemotherapy-induced IL8 upregulates MDR1/ABCB1 in tumor blood vessels and results in unfavorable outcome. Cancer Res.

[72] Kubiliute R., Januskeviciene I., Urbanaviciute R., Daniunaite K., Drobniene M., Ostapenko V. (2021). Nongenotoxic ABCB1 activator tetraphenylphosphonium can contribute to doxorubicin resistance in MX-1 breast cancer cell line. Sci Rep.

[73] Li L., Fridley B.L., Kalari K., Niu N., Jenkins G., Batzler A. (2014). Discovery of genetic biomarkers contributing to variation in drug response of cytidine analogues using human lymphoblastoid cell lines. BMC Genomics.

[74] Lindner A.U., Concannon C.G., Boukes G.J., Cannon M.D., Llambi F., Ryan D. (2013). Systems analysis of BCL2 protein family interactions establishes a model to predict responses to chemotherapy. Cancer Res.

[75] Wang C.C., Bajikar S.S., Jamal L., Atkins K.A., Janes K.A. (2014). A time- and matrix-dependent TGFBR3–JUND–KRT5 regulatory circuit in single breast epithelial cells and basal-like premalignancies. Nat Cell Biol.

[76] Leung E., Kannan N., Krissansen G.W., Findlay M.P., Baguley B.C. (2010). MCF-7 breast cancer cells selected for tamoxifen resistance acquire new phenotypes differing in DNA content, phospho-HER2 and PAX2 expression, and rapamycin sensitivity. Cancer Biol Ther.

[77] Franco G.R., Tanaka M., Simpson A.J., Pena S.D. (1998). Characterization of a *Schistosoma mansoni* homologue of the gene encoding the breast basic conserved protein 1/L13 ribosomal protein. Comp Biochem Physiol B Biochem Mol Biol.

[78] Zagidullin B., Aldahdooh J., Zheng S., Wang W., Wang Y., Saad J. (2019). DrugComb: an integrative cancer drug combination data portal. Nucleic Acids Res.

[79] Liu H., Zhang W., Zou B., Wang J., Deng Y., Deng L. (2020). DrugCombDB: a comprehensive database of drug combinations toward the discovery of combinatorial therapy. Nucleic Acids Res.

[80] Granger C.W.J. (1969). Investigating causal relations by econometric models and cross-spectral methods. Econometrica.

[81] Szklarczyk D., Gable A.L., Lyon D., Junge A., Wyder S., Huerta-Cepas J. (2019). STRING v11: protein–protein association networks with increased coverage, supporting functional discovery in genome-wide experimental datasets. Nucleic Acids Res.

[82] Ward J.H. (1963). Hierarchical grouping to optimize an objective function. J American Stat Assoc.

[83] Cairns J., Kalari K.R., Ingle J.N., Shepherd L.E., Ellis M.J., Goss P.E. (2021). Interaction between SNP genotype and efficacy of anastrozole and exemestane in early stage breast cancer. Clin Pharmacol Ther.

[84] Cairns J., Ingle J.N., Wickerham L.D., Weinshilboum R., Liu M., Wang L. (2017). SNPs near the cysteine proteinase cathepsin O gene (*CTSO*) determine tamoxifen sensitivity in ERα-positive breast cancer through regulation of BRCA1. PLoS Genet.

[85] Cairns J., Fridley B.L., Jenkins G.D., Zhuang Y., Yu J., Wang L. (2018). Differential roles of ERRFI1 in EGFR and AKT pathway regulation affect cancer proliferation. EMBO Rep.

[86] Cairns J., Ingle J.N., Dudenkov T.M., Kalari K.R., Carlson E.E., Na J. (2020). Pharmacogenomics of aromatase inhibitors in postmenopausal breast cancer and additional mechanisms of anastrozole action. JCI Insight.

[87] Cairns J., Ingle J.N., Kalari K.R., Shepherd L.E., Kubo M., Goetz M.P. (2019). The lncRNA MIR2052HG regulates ERα levels and aromatase inhibitor resistance through LMTK3 by recruiting EGR1. Breast Cancer Res.

