# Supplementary material for: Preclinical-to-clinical Anti-cancer Drug Response Prediction and Biomarker Identification Using TINDL
Source: Genomics Proteomics Bioinformatics. 2023 Feb 11;21(3):535–50. doi: 10.1016/j.gpb.2023.01.006 (PMC10787192; doi:10.1016/j.gpb.2023.01.006)
Supplement: Supplementary File S1 — Supplementary methods [file mmc1.docx]

**Supplementary methods**

**Adversarial Discriminative Domain Adaptation (ADDA)**

ADDA [1] is a unidirectional domain adaptation which takes a pretrained neural network and attempts to adapt the network to the target dataset ($T$) by forcing the latent feature space of the target datasets to be similar to that of the source dataset ($S$). First, a model is trained on the source dataset (CCLs in our case) until it converges or a certain performance metric is achieved. We then clone this model and train only the first $n$ layers of the cloned model (here first two layers), which is called the “target encoder” ($M{}_{t}$). Samples in the target dataset would pass through $M{}_{t}$, while samples from the source dataset would use the parameters of original model, which is called the “source encoder” ($M{}_{s}$). We then create third model, called the “discriminator” ($D$), which classifies whether the extracted features came from the source or the target datasets. This is trained using the following loss function:

$$\mathcal{L}_{D}=- \mathbb{E}{}_{X_{s}\sim S}[logD_{1}\left( M_{s}\left( X_{s} \right) \right)]- \mathbb{E}{}_{X_{t}\sim T}[\log D_{0}(M_{t}\left( X_{t} \right))].$$

(1)

where $D_{0}$ and $D_{1}$ are the outputs of the discriminator in first and second index, respectively. This pertains to the predicted probability of input from the target and source datasets and is constrained such that $D_{0}=1-D_{1}$.

Oppositely, the objective of the $M{}_{t}$ is to create representations that could confuse the discriminator, thus creating an adversarial optimization with the following loss:

$$\mathcal{L}_{M_{t}}=-\mathbb{E}{}_{X_{t}\sim T}[\log D_{1}(M_{t}\left( X_{t} \right))].$$

(2)

The target encoder and the discriminator are trained alternately until convergence or the maximum number of steps are achieved.

We used the TINDL models as the pretrained models and the first two layers as the feature extractors. The discriminator has one hidden layer with 128 neurons and two output neurons. We then selected the other hyperparameters (learning rate and number of training epochs) using 5-fold coefficient of variation (CV) where we used the maximum discriminator confusion as the criterion for selecting the hyperparameter.

**Domain Adaptive Neural Network (DANN)**

DANN [2] uses the shared latent feature space to allow the model to be used on the target dataset ($T$) despite only being trained using the labels of the source dataset ($S$). The model has three components: encoder $(M)$, predictor $(F)$, and discriminator$(D)$. The predictor is the model for the main task and it is trained using the appropriate loss function (*e.g.*, mean squared error for regression tasks, negative log-likelihood or cross entropy for classification tasks). The input of the predictor comes from the encoder, which extracts domain-invariant features from the data. The discriminator attempts to identify whether the extracted features came from the source or the target datasets. Ideally, the extracted features must have enough information for the main task, and at the same time confuse the discriminator. The model is trained using the following objective function:

$${\mathcal{L}= \mathbb{E}}_{\left( X_{s},y \right)\sim S}\left[ \left( F\left( M\left( X_{s} \right) \right)-y \right)^{2} \right]+{R_{\lambda}\mathcal{(L}}_{D}).$$

(3)

Here, $\mathcal{L}_{D}(\cdot)$ is the classification loss of the discriminator, which is given by:

$$\mathcal{L}_{D}=- \mathbb{E}{}_{X_{s}\sim S}[logD_{1}\left( M\left( X_{s} \right) \right)]- \mathbb{E}{}_{X_{t}\sim T}[\log\left( D_{0}\left( M\left( X_{t} \right) \right) \right],$$

(4)

where $D_{0}$ and $D_{1}$ are the outputs of the discriminator in first and second index, respectively. This pertains to the predicted probability of input from the target and source datasets and is constrained such that $D_{0}=1-D_{1}$. A gradient reversal technique is used on the discriminator loss to allow end-to-end training and is denoted by $R_{\lambda}$ in objective function. $R_{\lambda}$ does not affect the network during forward propagation, but $R_{\lambda}$ negates the gradients during backward pass.

We used the TINDL models as the pretrained models and the first two layers as the feature extractors. The discriminator has one hidden layer with 128 neurons and two output neurons. We then selected the other hyperparameters using 5-fold CV as described in the Methods section.

**Kaplan–Meier survival analysis**

An online database Kaplan–Meier Plotter (<http://kmplot.com/analysis/>) [3] was used to investigate the association between *RPS6* and *RPL13* messenger RNA (mRNA) levels and survival of estrogen receptor positive/HER2 negative breast cancer patients. The patients in datasets were classified into two groups according to auto select best cutoff (high *vs.* low expression). The hazard ratio (HR) with 95 % confidence intervals (CI) and log-rank *P* value were calculated and shown on the web pages. *P* value of < 0.05 was considered to be statistically significant.

**References**

[1] Tzeng E, Hoffman J, Saenko K, Darrell T. Adversarial Discriminative Domain Adaptation. IEEE Conf Comput Vis Pattern Recognit 2017:7167–76.

[2] Ganin Y, Lempitsky V. Unsupervised domain adaptation by backpropagation. Proc 32nd Int Conf Mach Learn 2015:1180–9.

[3] Nagy A, Munkacsy G, Gyorffy B. Pancancer survival analysis of cancer hallmark genes. Sci Rep 2021;11:6047.
