# Supplementary figures and images for: Preclinical-to-clinical Anti-cancer Drug Response Prediction and Biomarker Identification Using TINDL
Source: Genomics Proteomics Bioinformatics. 2023 Feb 11;21(3):535–50. doi: 10.1016/j.gpb.2023.01.006 (PMC10787192; doi:10.1016/j.gpb.2023.01.006)

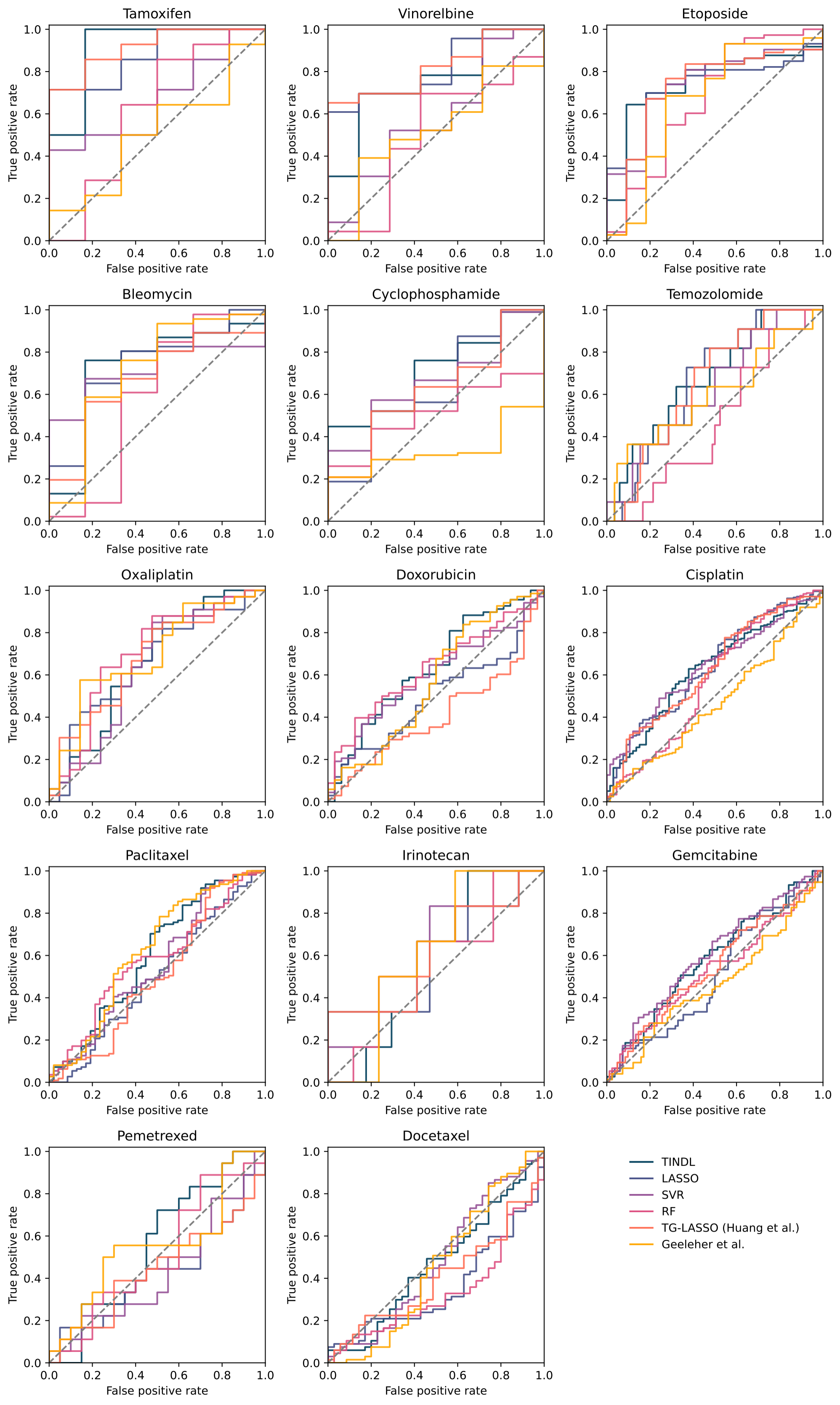

Supplement: Supplementary Figure S1 — ROC curves of TINDL and baseline approaches in different drugs LASSO, least absolute shrinkage and selection operator; SVR, support vector regression; RF, random forest; TG-LASSO, tissue guided LASSO [file mmc2.pdf]

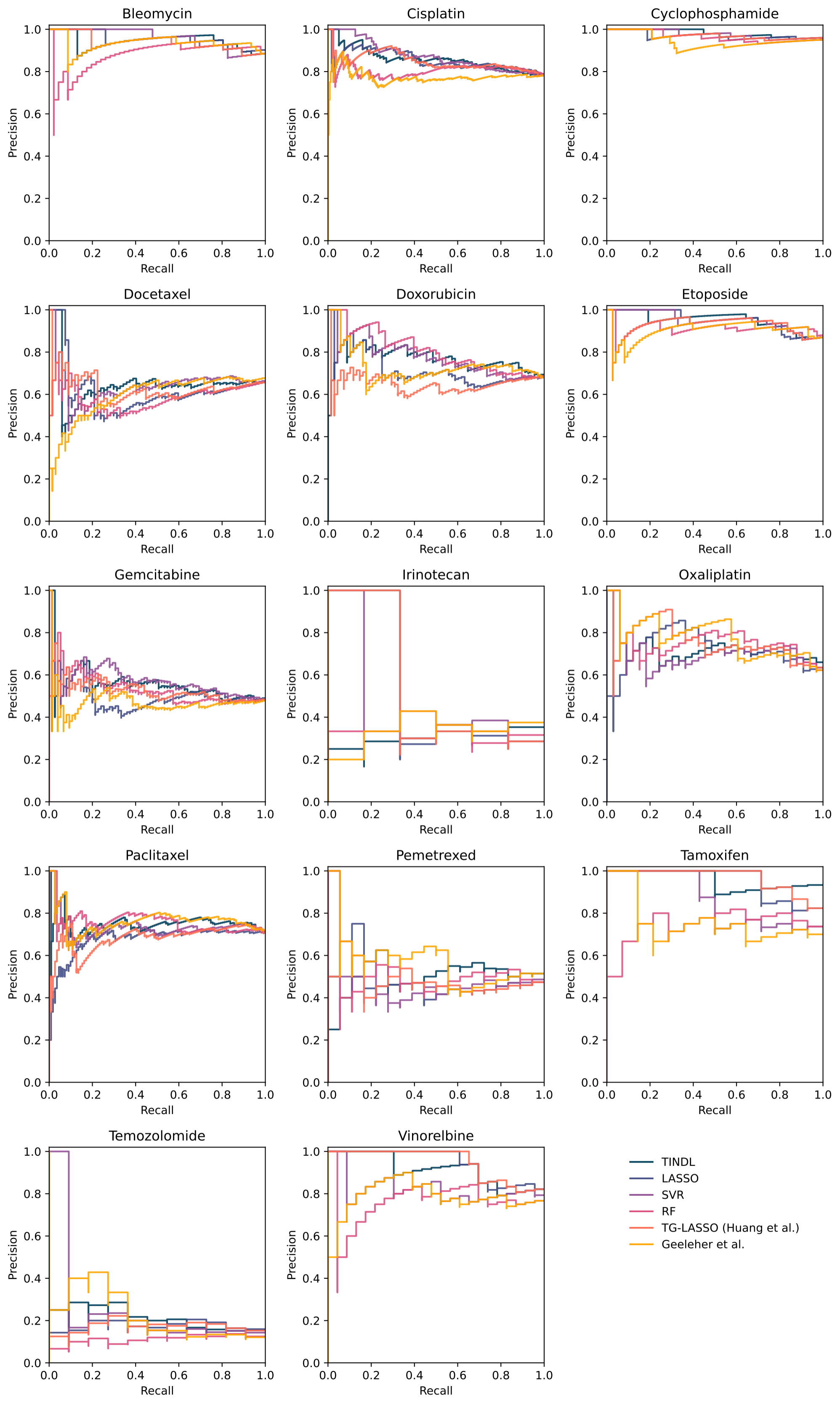

Supplement: Supplementary Figure S2 — Precision–recall curves of TINDL and baseline approaches in different drugs [file mmc3.pdf]

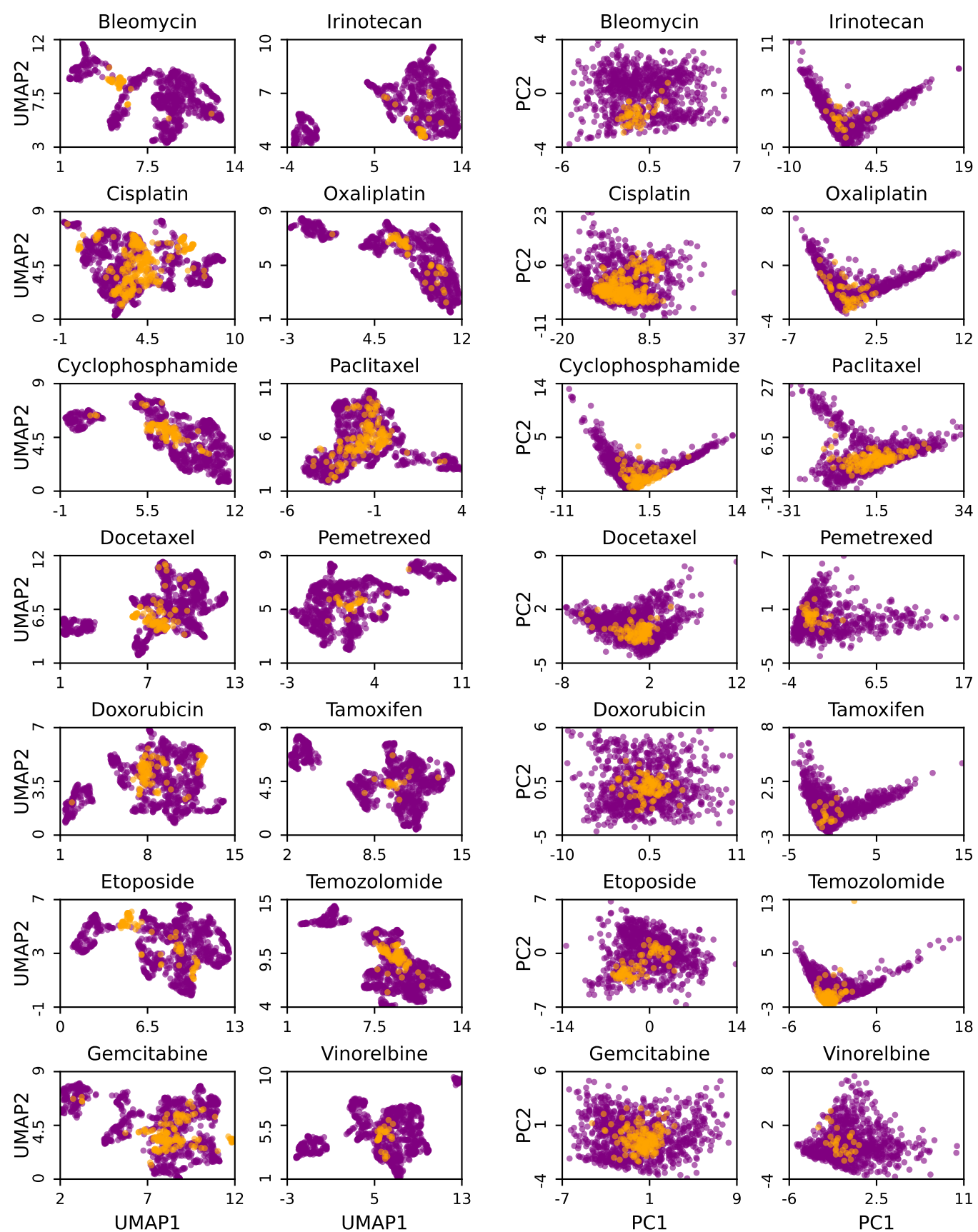

Supplement: Supplementary Figure S3 — UMAP and PCA plots of the learned latent features of different drugs by TINDL Purple points indicate cell line samples (GDSC) and orange points indicate tumor samples (TCGA). UMAP, Uniform Manifold Approximation and Projection. [file mmc4.pdf]

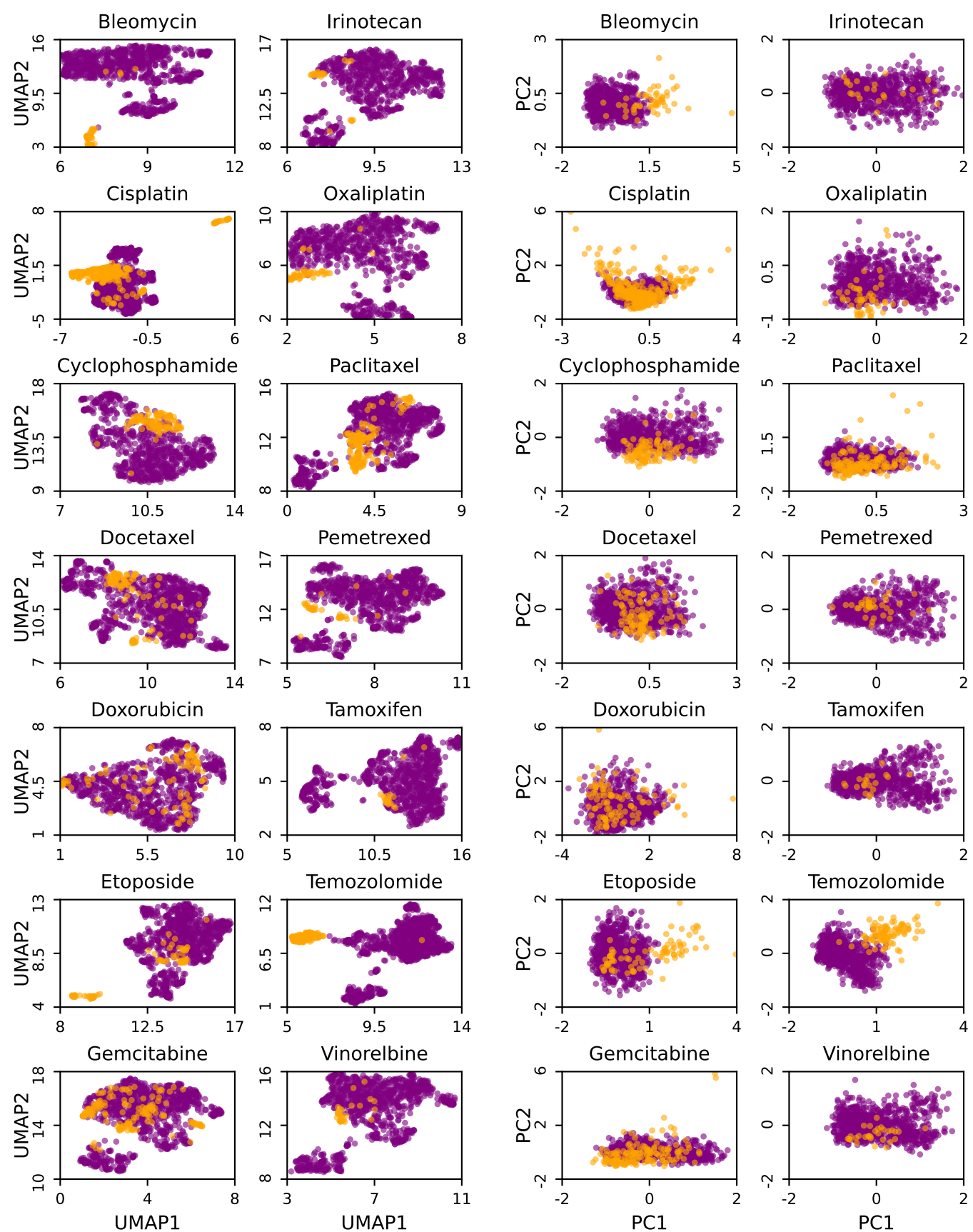

Supplement: Supplementary Figure S4 — UMAP and PCA plots of the learned latent features of different drugs by Combat-DL Purple points indicate cell line samples (GDSC) and orange points indicate tumor samples (TCGA). [file mmc5.pdf]

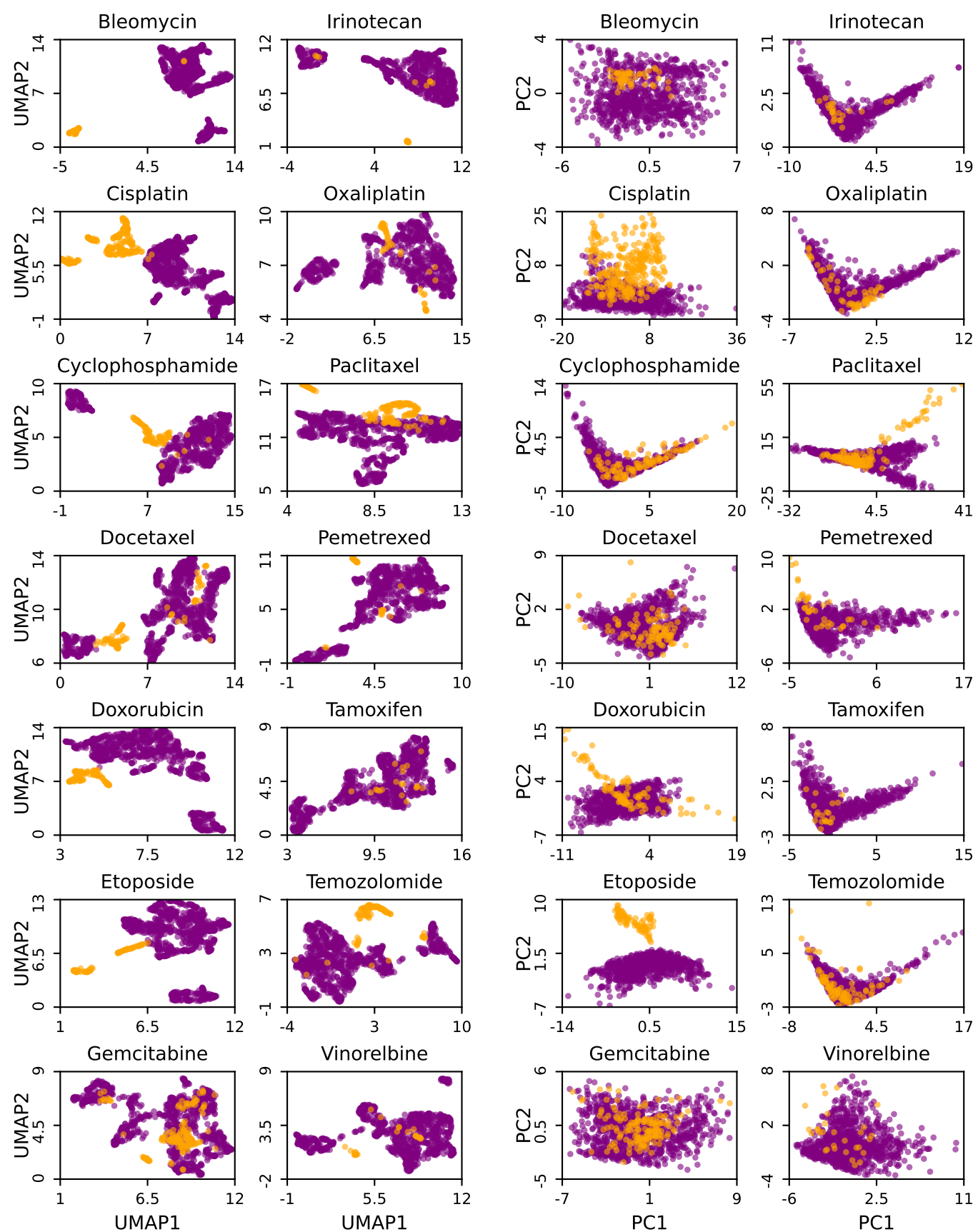

Supplement: Supplementary Figure S5 — UMAP and PCA plots of the learned latent features of different drugs by ADDA-DL Purple points indicate cell line samples (GDSC) and orange points indicate tumor samples (TCGA). [file mmc6.pdf]

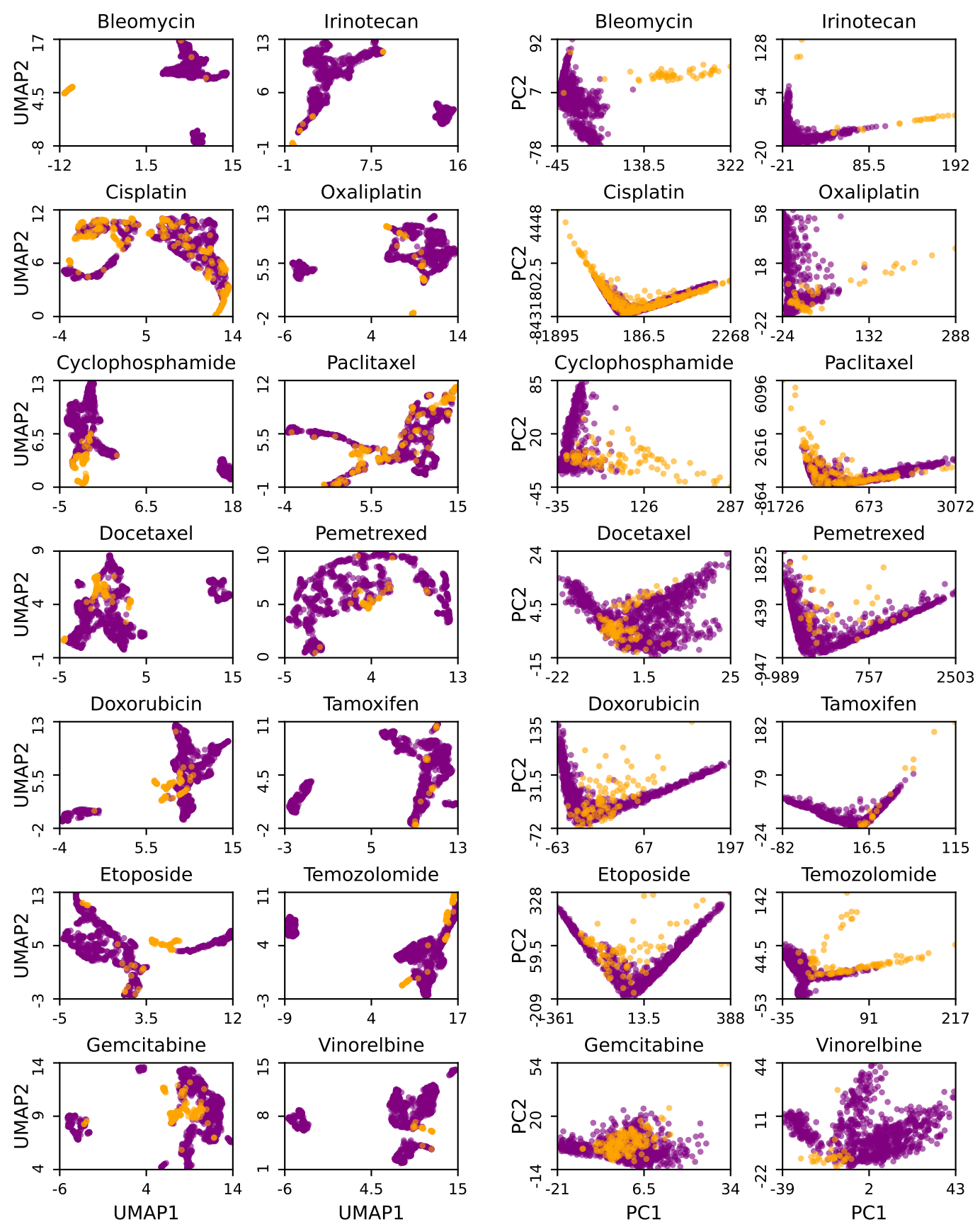

Supplement: Supplementary Figure S6 — UMAP and PCA plots of the learned latent features of different drugs by DANN-DL Purple points indicate cell line samples (GDSC) and orange points indicate tumor samples (TCGA). [file mmc7.pdf]

**A**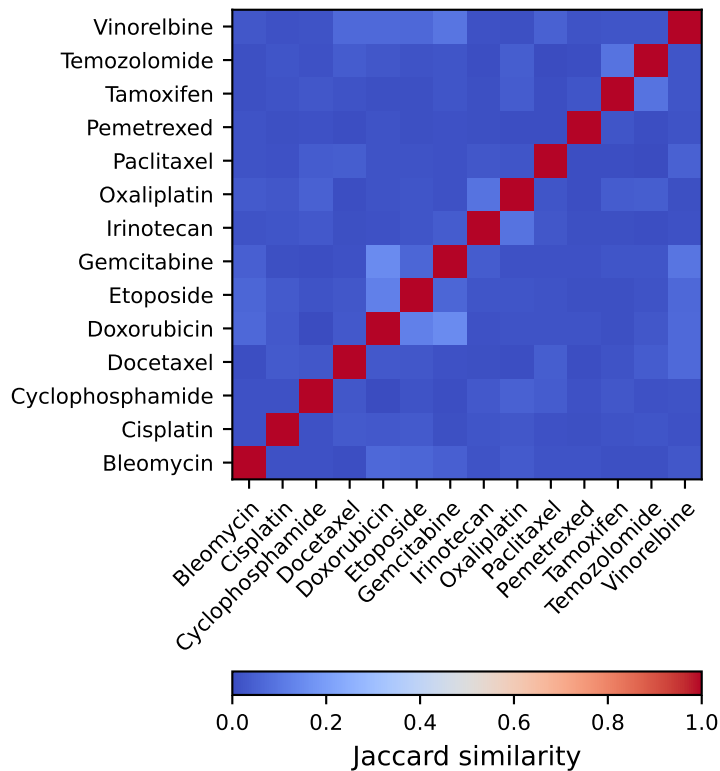**B**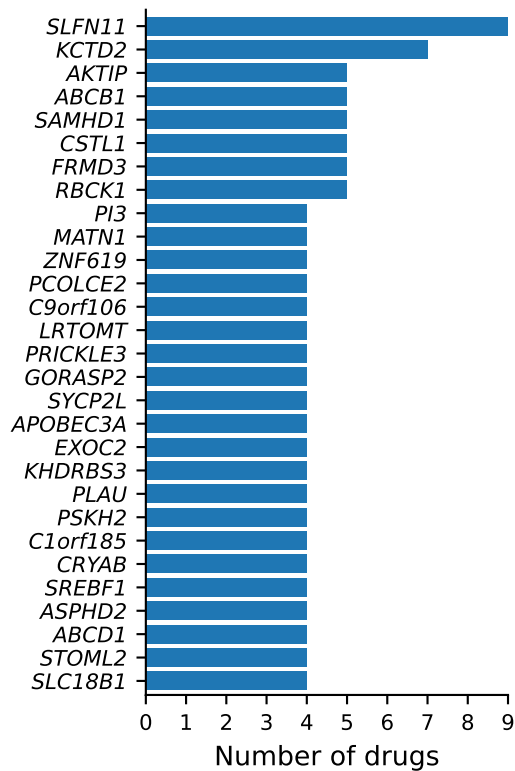

Supplement: Supplementary Figure S8 — Genes identified by TINDL for different drugs A. Heatmap of the Jaccard similarity of the selected top genes in the 14 drugs. B. Number of drugs in which the genes were identified as a top gene. Only genes that were present in the top genes of at least four drugs are included. [file mmc9.pdf]

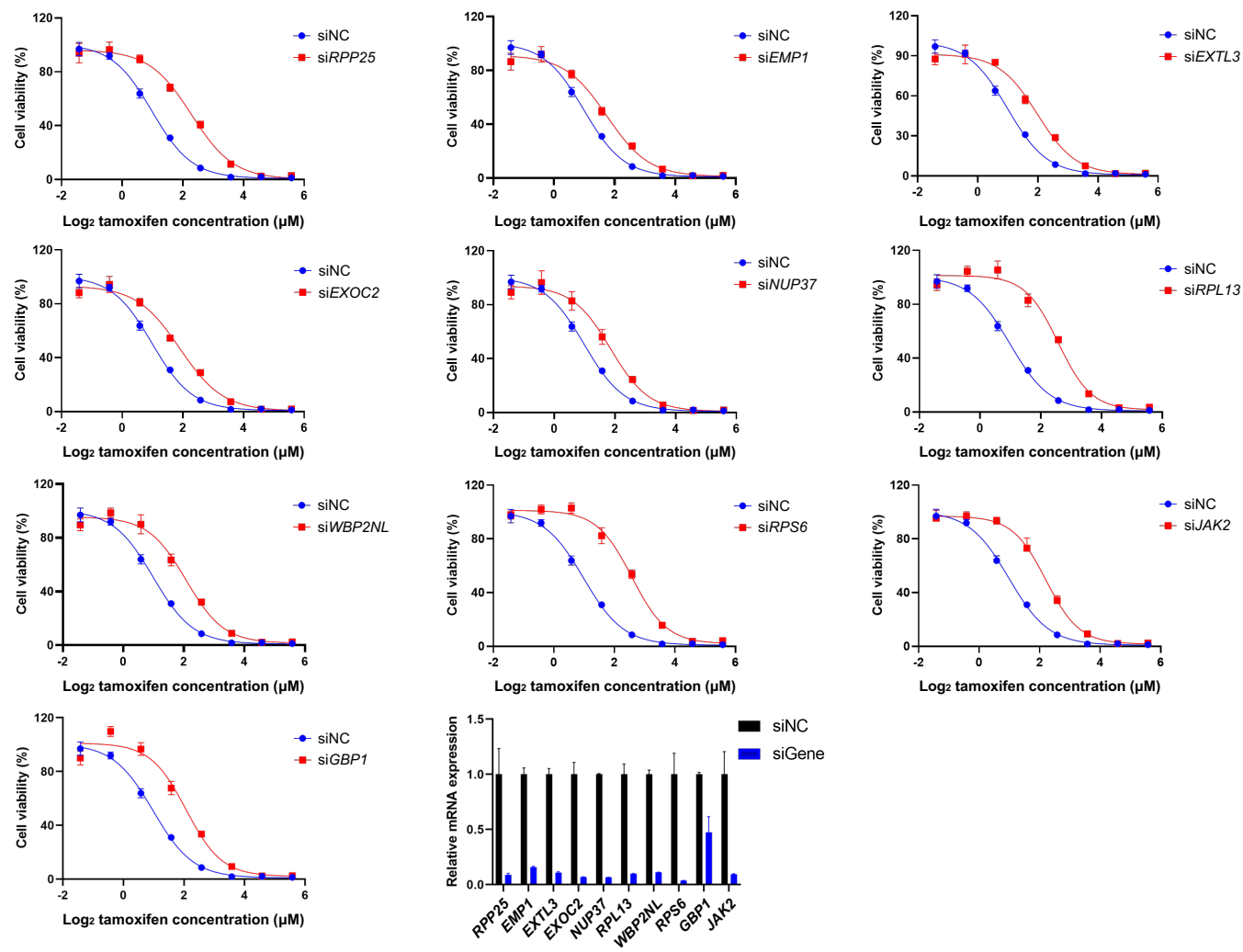

Supplement: Supplementary Figure S9 — Tamoxifen dose–response curves corresponding to the siRNA knockdown of 10 genes identified by TINDL in MCF7 cells Knockdown efficiency was assessed by qRT-PCR using three technical replicates (Table S8). Gene expression was normalized to siRNA negative control. Table S8 shows the knockdown efficiency of each gene and corresponding statistical analysis. [file mmc10.pdf]

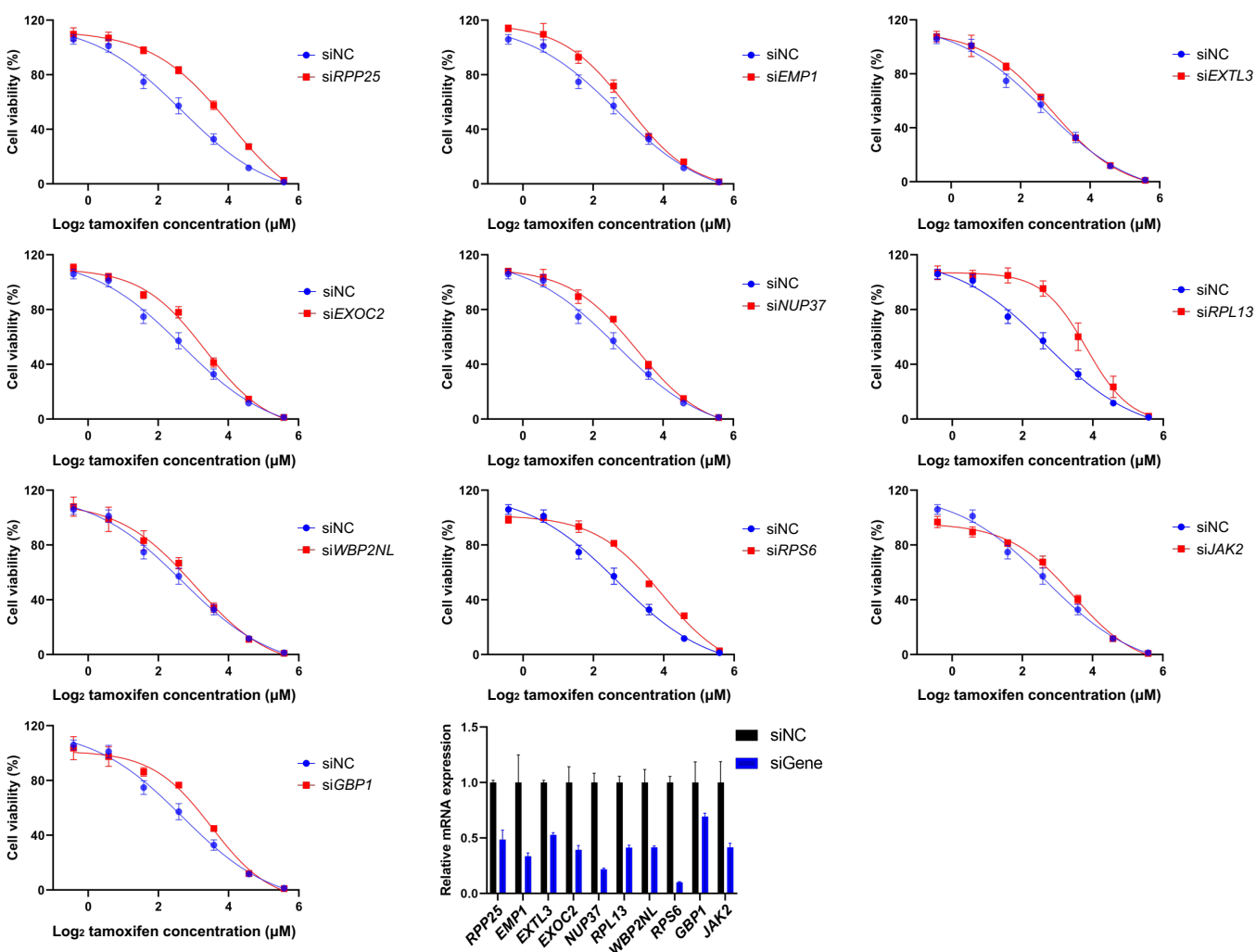

Supplement: Supplementary Figure S10 — Tamoxifen dose–response curves corresponding to the siRNA knockdown of 10 genes identified by TINDL in T47D cells Knockdown efficiency was assessed by qRT-PCR using three technical replicates (Table S8). Gene expression was normalized to siRNA negative control. Table S8 shows the knockdown efficiency of each gene and corresponding statistical analysis. [file mmc11.pdf]

A

RPS6 (200081\_s\_at)

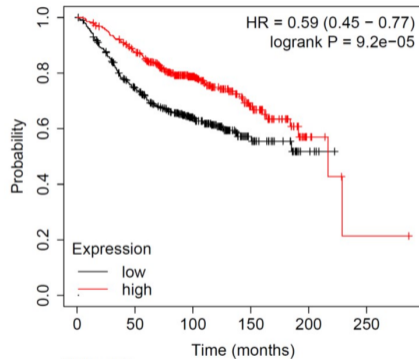

B

RPS6 (200081\_s\_at)

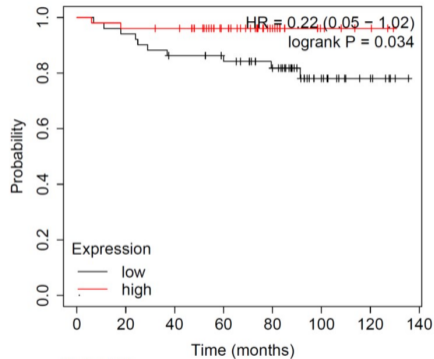

C

RPL13 (214976\_at)

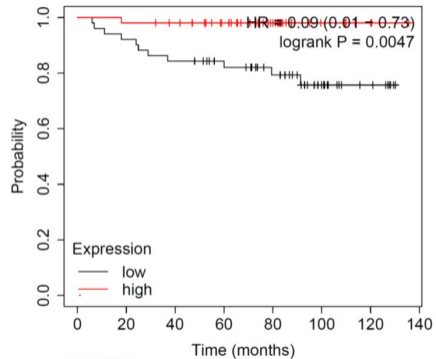

Supplement: Supplementary Figure S11 — Kaplan–Meier survival analysis of RPS6 and RPL13 gene expression in estrogen receptor positive/HER2 negative breast cancer patients using Kaplan–Meier Plotter A. RFS of RPS6 in systemically untreated patients. B. RFS of RPS6 in tamoxifen treated patients. C. RFS of RPL13 in tamoxifen treated patients. RFS, relapse free survival; HR, hazard ratio. [file mmc12.pdf]

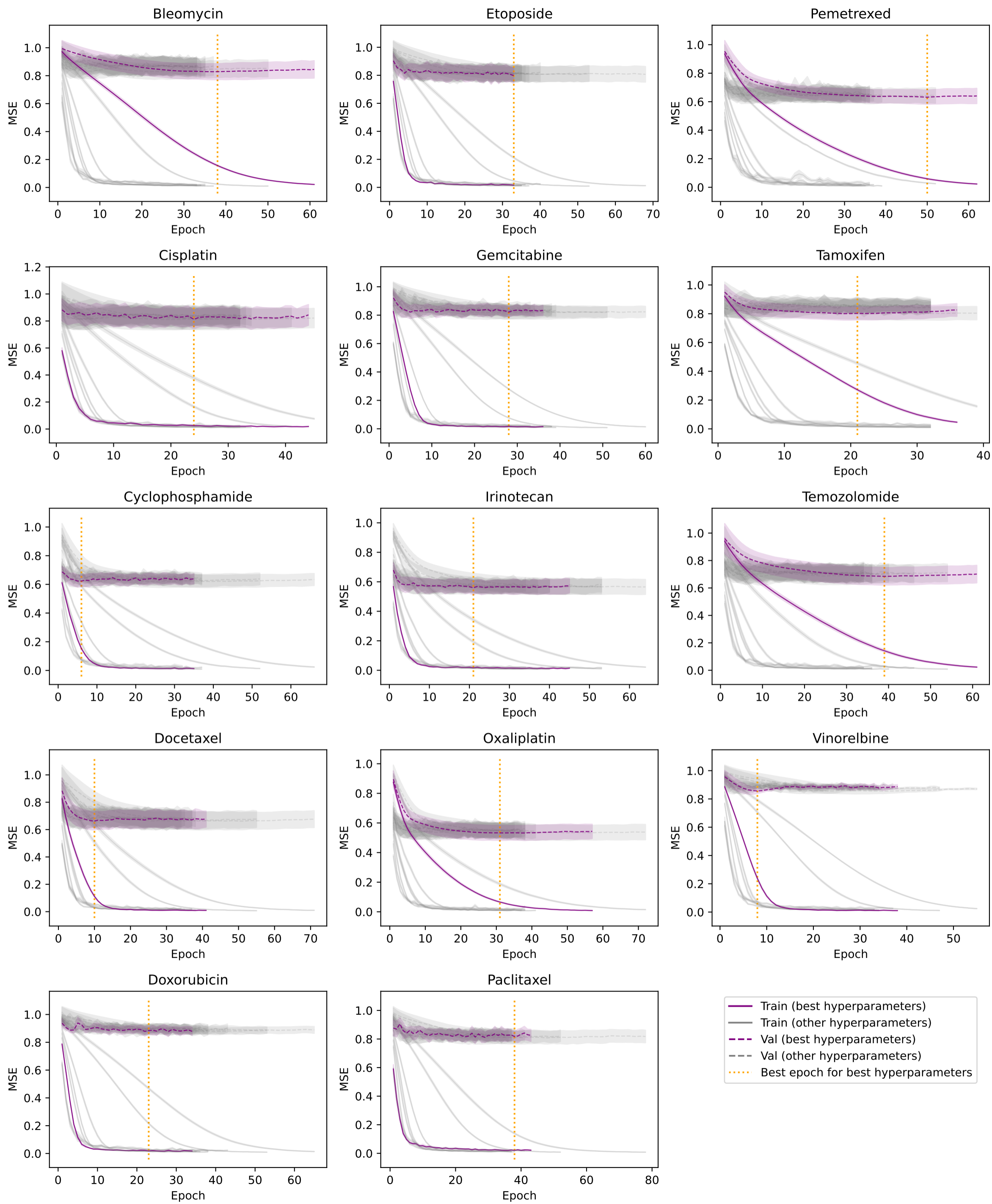

Supplement: Supplementary Figure S12 — MSE curves of different hyperparameters during grid-search To reduce cluttering, only the average value across the five folds were plotted. The shaded regions are the 90% confidence intervals. The chosen number of epochs is denoted as a vertical line, while the purple curves represent the training and validation curves of the chosen set of hyperparameters. MSE, mean squared error. [file mmc13.pdf]

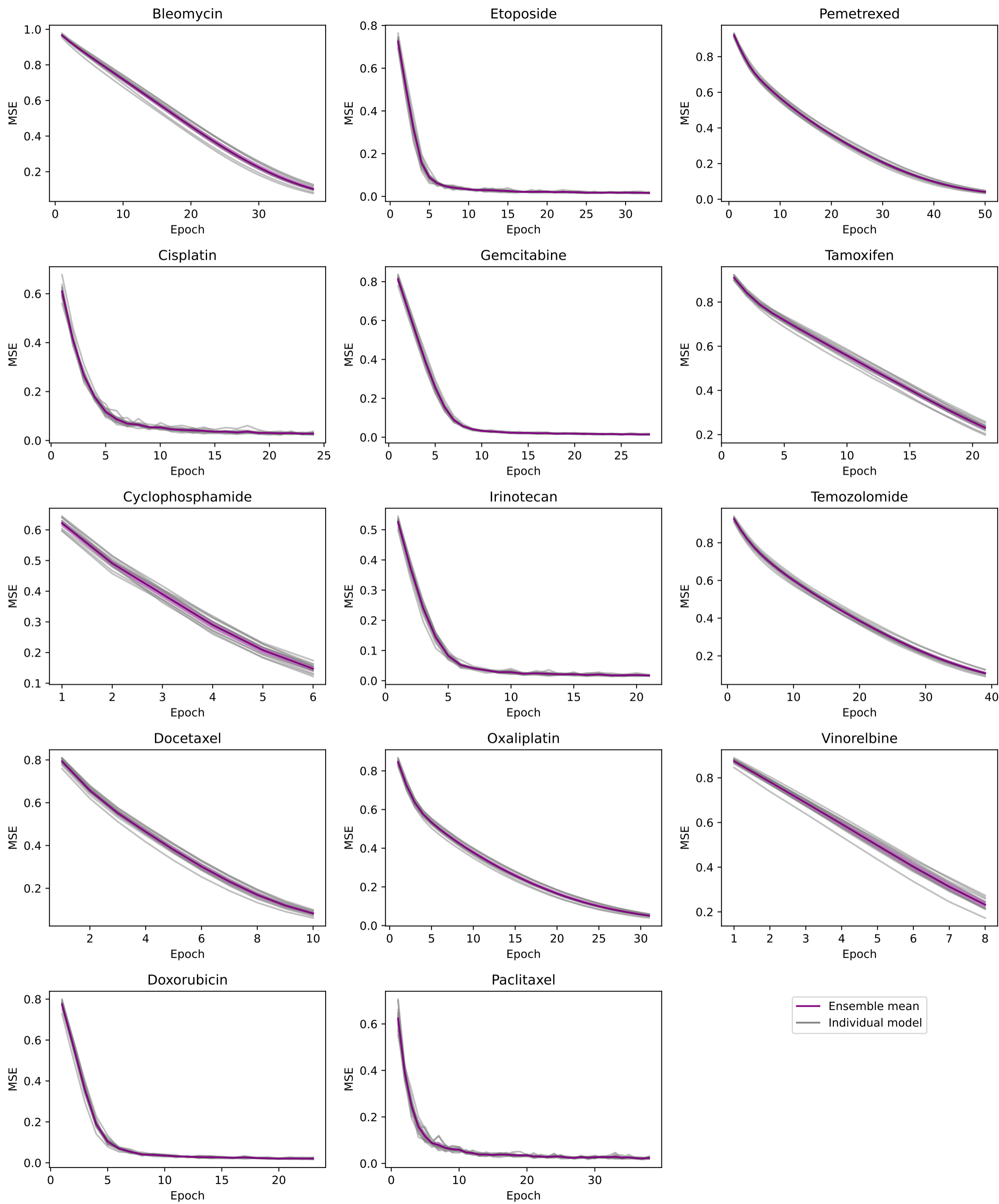

Supplement: Supplementary Figure S13 — Training MSE curves of the 10 different initializations during the final training Individual models are denoted as the gray curves while their average is denoted by the purple curve. The final training uses all the labeled cell lines as the training set so there are no validation curves. [file mmc14.pdf]
