# Supplementary material for: Preclinical-to-clinical Anti-cancer Drug Response Prediction and Biomarker Identification Using TINDL
Source: Genomics Proteomics Bioinformatics. 2023 Feb 11;21(3):535–50. doi: 10.1016/j.gpb.2023.01.006 (PMC10787192; doi:10.1016/j.gpb.2023.01.006)
Supplement: Supplementary Figure S7 — Contribution scores of genes in the trained model of TINDL The Y-axis shows the contribution score, and the X-axis shows genes in a descending order of their score. Orange points indicate the “knees” found by kneedle algorithm, representing the threshold below which the contribution of genes to the trained model is small. The numbers in the brackets show number of genes above threshold and threshold. [file mmc8.pdf]

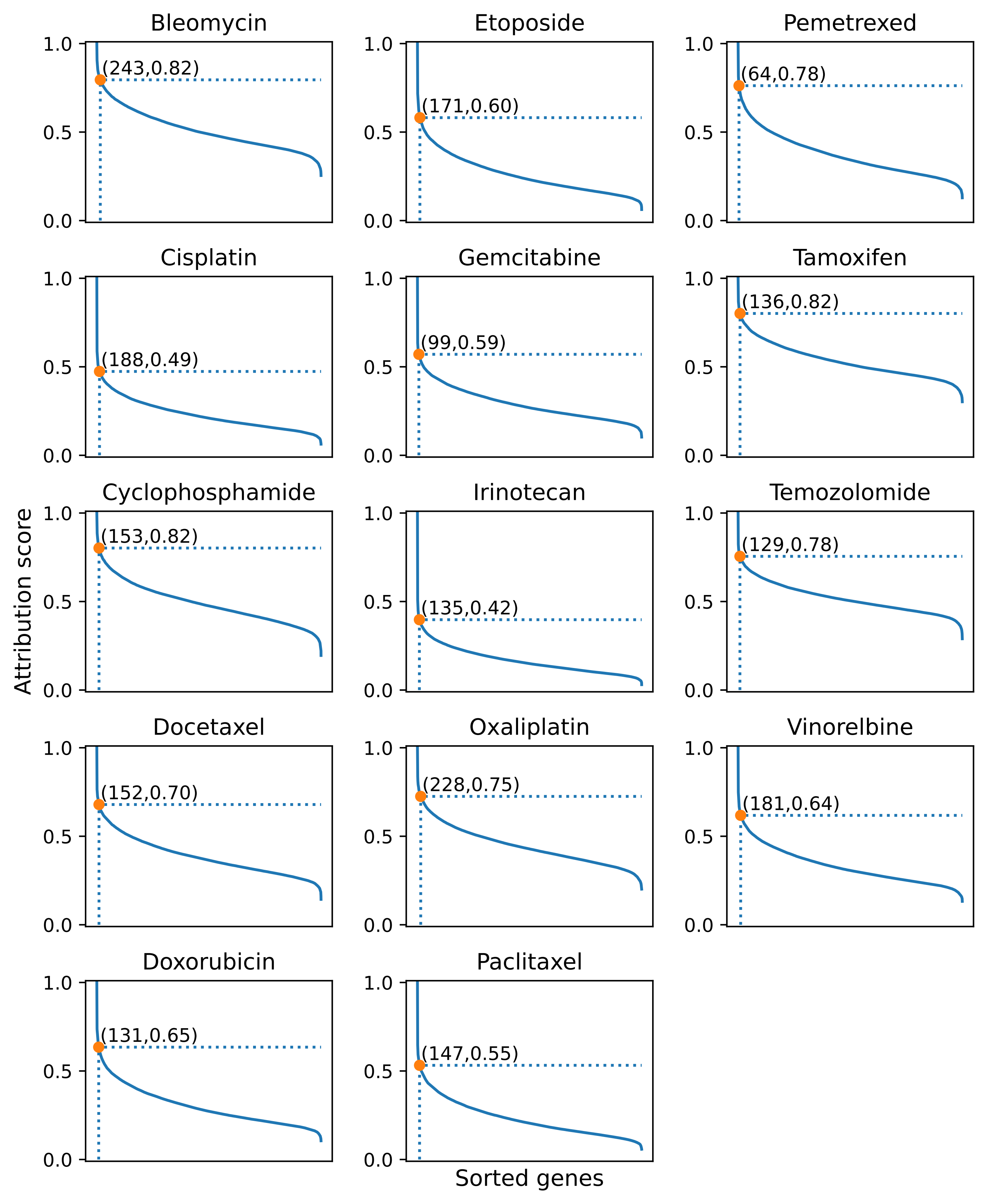
Bleomycin
(243,0.82)
Etoposide
(171,0.60)
Pemetrexed
(64,0.78)
Cisplatin
(188,0.49)
Gemcitabine
(99,0.59)
Tamoxifen
(136,0.82)
Cyclophosphamide
(153,0.82)
Irinotecan
(135,0.42)
Temozolomide
(129,0.78)
Docetaxel
(152,0.70)
Oxaliplatin
(228,0.75)
Vinorelbine
(181,0.64)
Doxorubicin
(131,0.65)
Paclitaxel
(147,0.55)
Attribution score
Sorted genes
0.0
0.5
1.0
