# Supplementary material for: Preclinical-to-clinical Anti-cancer Drug Response Prediction and Biomarker Identification Using TINDL
Source: Genomics Proteomics Bioinformatics. 2023 Feb 11;21(3):535–50. doi: 10.1016/j.gpb.2023.01.006 (PMC10787192; doi:10.1016/j.gpb.2023.01.006)
Supplement: Supplementary Table S3 — Precision at kth percentile of TINDL [file mmc17.docx]

**Table S3 Precision at k^th^ percentile of TINDL**

| **Drug** | **k = 10** | **k = 20** | **k = 30** | **k = 40** | **k = 50** |
| --- | --- | --- | --- | --- | --- |
| Bleomycin | 1.000 | 0.909 | 0.938 | 0.952 | 0.962 |
| Cisplatin | 0.935 | 0.885 | 0.868 | 0.843 | 0.862 |
| Cyclophosphamide | 1.000 | 1.000 | 1.000 | 1.000 | 0.980 |
| Docetaxel | 0.455 | 0.619 | 0.645 | 0.659 | 0.647 |
| Doxorubicin | 0.800 | 0.800 | 0.833 | 0.800 | 0.780 |
| Etoposide | 1.000 | 0.941 | 0.960 | 0.971 | 0.976 |
| Gemcitabine | 0.625 | 0.563 | 0.553 | 0.571 | 0.544 |
| Irinotecan | 0.000 | 0.200 | 0.286 | 0.222 | 0.333 |
| Oxaliplatin | 0.667 | 0.727 | 0.688 | 0.727 | 0.741 |
| Paclitaxel | 0.688 | 0.719 | 0.771 | 0.730 | 0.759 |
| Pemetrexed | 0.250 | 0.625 | 0.417 | 0.467 | 0.526 |
| Tamoxifen | 1.000 | 1.000 | 1.000 | 0.875 | 0.900 |
| Temozolomide | 0.200 | 0.211 | 0.172 | 0.184 | 0.167 |
| Vinorelbine | 1.000 | 1.000 | 0.889 | 0.917 | 0.933 |

*Note*: This measure captures the ability of the model to identify sensitive patients based on different thresholds, determined based on the value of k. Detailed procedure for this calculation is provided in the Methods section.
