# Supplementary material for: Preclinical-to-clinical Anti-cancer Drug Response Prediction and Biomarker Identification Using TINDL
Source: Genomics Proteomics Bioinformatics. 2023 Feb 11;21(3):535–50. doi: 10.1016/j.gpb.2023.01.006 (PMC10787192; doi:10.1016/j.gpb.2023.01.006)
Supplement: Supplementary Table S7 — The result of siRNA gene knockdown experiments in MCF7 and T47D cell lines for 10 genes identified by TINDL for tamoxifen [file mmc21.docx]

Table S7 The result of siRNA gene knockdown experiments in MCF7 and T47D cell lines for 10 genes identified by TINDL for tamoxifen

| **Gene** | **Rank by TINDL** | **MCF7** | | **T47D** | |
| --- | --- | --- | --- | --- | --- |
|  |  | ***P* value** | **IC50 Change** | ***P* value** | **IC50 Change** |
| *RPP25* | 1 | <0.0001 | 146% | <0.0001 | 158% |
| *EMP1* | 2 | <0.0001 | 69% | 0.08 | 23% |
| *EXTL3* | 3 | <0.0001 | 101% | 0.16 | 17% |
| *EXOC2* | 4 | <0.0001 | 89% | 0.0007 | 58% |
| *NUP37* | 5 | <0.0001 | 83% | 0.0016 | 48% |
| *RPL13* | 6 | <0.0001 | 201% | 0.0011 | 119% |
| *WBP2NL* | 7 | <0.0001 | 113% | 0.09 | 29% |
| *RPS6* | 8 | <0.0001 | 202% | <0.0001 | 141% |
| *GBP1* | 9 | <0.0001 | 113% | 0.0012 | 78% |
| *JAK2* | 19 | <0.0001 | 134% | 0.0002 | 70% |

*Note*: The *P* values are calculated using an extra sum-of-squares F test. Genes are sorted based on their rank by TINDL. siRNA, small interfering RNA.
