# Supplementary material for: Preclinical-to-clinical Anti-cancer Drug Response Prediction and Biomarker Identification Using TINDL
Source: Genomics Proteomics Bioinformatics. 2023 Feb 11;21(3):535–50. doi: 10.1016/j.gpb.2023.01.006 (PMC10787192; doi:10.1016/j.gpb.2023.01.006)
Supplement: Supplementary Table S9 — Learning rates, and batch sizes, and number of epochs used in the final models [file mmc23.docx]

**Table S9 Learning rates, and batch sizes, and number of epochs used in the final models**

| **Drug** | **Learning rate** | **Batch size** | **Number of epochs** |
| --- | --- | --- | --- |
| Bleomycin | 0.00001 | 128 | 38 |
| Cisplatin | 0.0005 | 128 | 24 |
| Cyclophosphamide | 0.0001 | 128 | 6 |
| Docetaxel | 0.00005 | 64 | 10 |
| Doxorubicin | 0.0001 | 64 | 23 |
| Etoposide | 0.0001 | 64 | 33 |
| Gemcitabine | 0.00005 | 64 | 28 |
| Irinotecan | 0.0001 | 64 | 21 |
| Oxaliplatin | 0.00001 | 64 | 31 |
| Paclitaxel | 0.0005 | 64 | 38 |
| Pemetrexed | 0.00001 | 128 | 50 |
| Tamoxifen | 0.00001 | 64 | 21 |
| Temozolomide | 0.00001 | 128 | 39 |
| Vinorelbine | 0.00005 | 128 | 8 |

*Note*: These hyperparameters were selected using 5-fold cross validations on the CCL samples.
